# Fundamentally Manipulating the Electronic Structure of Polar Bifunctional Catalysts for Lithium‐Sulfur Batteries: Heterojunction Design versus Doping Engineering

**DOI:** 10.1002/advs.202307995

**Published:** 2024-03-11

**Authors:** Huifang Xu, Qingbin Jiang, Zheng Shu, Kwan San Hui, Shuo Wang, Yunshan Zheng, Xiaolu Liu, Huixian Xie, Weng‐Fai (Andy) Ip, Chenyang Zha, Yongqing Cai, Kwun Nam Hui

**Affiliations:** ^1^ Joint Key Laboratory of the Ministry of Education Institute of Applied Physics and Materials Engineering University of Macau Avenida da Universidade Taipa Macau SAR China; ^2^ School of Engineering Faculty of Science University of East Anglia Norwich NR4 7TJ UK; ^3^ Department of Physics and Chemistry Faculty of Science and Technology University of Macau Macau 999078 China

**Keywords:** active sites, doping strategies, electronic structures, heterogeneous structures, polar catalysts

## Abstract

Heterogeneous structures and doping strategies have been intensively used to manipulate the catalytic conversion of polysulfides to enhance reaction kinetics and suppress the shuttle effect in lithium‐sulfur (Li‐S) batteries. However, understanding how to select suitable strategies for engineering the electronic structure of polar catalysts is lacking. Here, a comparative investigation between heterogeneous structures and doping strategies is conducted to assess their impact on the modulation of the electronic structures and their effectiveness in catalyzing the conversion of polysulfides. These findings reveal that Co_0.125_Zn_0.875_Se, with metal‐cation dopants, exhibits superior performance compared to CoSe_2_/ZnSe heterogeneous structures. The incorporation of low Co^2+^ dopants induces the subtle lattice strain in Co_0.125_Zn_0.875_Se, resulting in the increased exposure of active sites. As a result, Co_0.125_Zn_0.875_Se demonstrates enhanced electron accumulation on surface Se sites, improved charge carrier mobility, and optimized both *p*‐band and *d*‐band centers. The Li‐S cells employing Co_0.125_Zn_0.875_Se catalyst demonstrate significantly improved capacity (1261.3 mAh g^−1^ at 0.5 C) and cycle stability (0.048% capacity delay rate within 1000 cycles at 2 C). This study provides valuable guidance for the modulation of the electronic structure of typical polar catalysts, serving as a design directive to tailor the catalytic activity of advanced Li‐S catalysts.

## Graphical abstract

A comparative study is conducted between widely explored heterogeneous structures and doping strategies to investigate their impact on the modulation of polar catalyst electronic structures and their effectiveness in catalyzing the conversion of polysulfides. The results show that doping strategies exhibit a more pronounced bidirectional catalytic effect of Li_2_S and facilitate rapid anchoring of polysulfides.

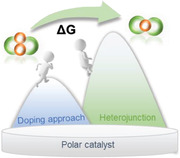

## 1 Introduction

The growing demand for high‐energy density and long‐cycle‐life rechargeable batteries, driven by the rapid development of electric vehicles and smart power grids, has brought lithium‐sulfur (Li‐S) batteries into the limelight.^[^ ^1^, ^2^ ^]^ Li‐S batteries utilize sulfur as the cathode and lithium metal as the anode, offering remarkable merits, including a high theoretical specific capacity of 1672 and 3860 mAh g^−1^ for sulfur and lithium, respectively.^[^ ^3^ ^]^ The combination of these high‐capacity electrodes culminates in an extraordinarily high theoretical energy density of 2600 Wh kg^−1^, surpassing that of commercially available battery systems. Additionally, sulfur, being an environmentally friendly and abundantly available element, enhances the potential cost‐effectiveness of Li‐S batteries.^[^ ^4^, ^5^, ^6^, ^7^, ^8^ ^]^ However, despite these intrinsic advantages, some knotty hurdles, such as the complicated multiphase transformation processes and the inherent nature of sulfur species, impede the commercialization of Li‐S batteries.^[^ ^9^, ^10^ ^]^ The low conductivity of sulfur leads to low sulfur utilization and large polarization due to the increased battery internal resistance and sluggish reaction kinetics. Furthermore, the discharge products (Li_2_S_2_ and Li_2_S) also possess inadequate conductivity and tend to accumulate on the surface of active materials, impeding electron and ion transportation and reducing sulfur utilization.^[^ ^11^, ^12^, ^13^ ^]^ Another significant challenge arises from the high energy barrier associated with the complete conversion of Li_2_S, as this process entails a phase transition involving the growth and nucleation of solid discharging products, ultimately leading to sluggish electrochemical kinetics.^[^ ^14^, ^15^ ^]^ Thirdly, the dissolution of intermediates, known as lithium polysulfides (LiPSs), induces a “shuttle effect” during cycling, resulting in low Coulombic efficiency and poor electrochemical cyclability of Li‐S batteries.^[^ ^16^ ^]^ Moreover, the situation is exacerbated with increased areal sulfur loading.^[^ ^17^, ^18^ ^]^

In the past few years, the mainstream strategy has been the use of various polar materials with an adsorptive effect to chemically bind LiPSs and facilitate the Li_2_S redox reaction, mitigating the shuttle effect,^[^ ^19^, ^20^, ^21^ ^]^ including metal oxides,^[^ ^22^, ^23^ ^]^ sulfides,^[^ ^24^, ^25^ ^]^ selenides,^[^ ^26^, ^27^ ^]^ and nitrides^[^ ^28^ ^]^), etc.^[^ ^21^, ^29^ ^]^ Among these materials, metal selenides with polar characteristics and high electrical conductivity have drawn increasingly extensive research interests for energy storage and conversion in recent years.^[^ ^30^, ^31^ ^]^ Unfortunately, once the polar active sites are encapsulated by LiPSs, the accumulated LiPSs continue to diffuse and gather in the electrolyte due to seriously sluggish reaction kinetics. Hence, strategies focused on polar electrocatalysts to thoroughly investigate the regulation of polysulfide kinetics, including LiPS conversion, Li_2_S precipitation/decomposition, and Li‐ion diffusion, are required.^[^ ^32^, ^33^, ^34^ ^]^ Heterogeneous structure design and doping engineering are introduced as two main approaches to fundamentally manipulate the electronic structure of polar materials. These approaches can enhance the mobility of charge carriers, optimize the *d*‐band center, create additional active sites, and fine‐tune the energetics and kinetics of catalytic reactions, thus leading to some unprecedented properties.^[^ ^35^, ^36^, ^37^ ^]^ For example, Chu et al. successfully engineered a heterostructure that combines the merits of strong adsorption (Co_3_O_4_) and high catalytic activities of materials (CoSe_2_).^[^ ^38^ ^]^ TheLiPSs adhering to the Co_3_O_4_ interface facilitate migration toward the CoSe_2_ region, thereby serving as an effective means to mitigate the shuttle effect and enhance the utilization of sulfur. Shen et al. synthesized a series of cations (Mn^2+^, Fe^2+^, Co^2+^, Ni^2+^, or Cu^2+^) doped ZnS catalysts by substituting cations of a parent ZnS lattice. These 3d dopants have the capability to alter the *d*‐band centers of active sites and fine‐tune their interaction with the frontier orbitals of polysulfides.^[^ ^39^ ^]^ These studies have demonstrated that introducing heteroatom‐doping and the unique interface of heterostructures represents effective approaches for modifying polar catalysts to enhance the adsorption effect and catalytic activities towards LiPSs and Li_2_S.^[^ ^40^, ^41^ ^]^ Nevertheless, there is a limited number of publications investigating the modulation of intrinsic electronic structures in metal selenides and their subsequent effect on catalytic activity through doping engineering or heterogeneous structure design. Specifically, a comprehensive comparison between these two effective strategies, which can provide profound insights into the design of polar electrocatalysts for Li‐S batteries, is a crucial but unexplored direction in this field.

**Figure 1:**
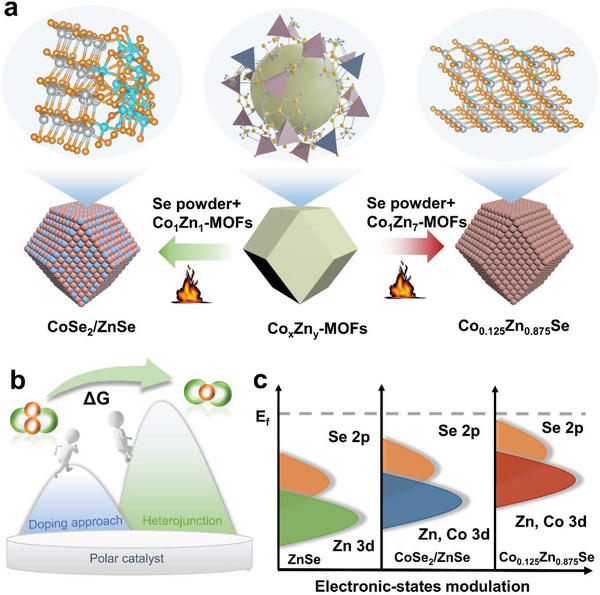
a) Illustrations of the synthesis route of doping engineering and heterojunction design catalytic. b) Schematic of doping engineering with better selectivity for Li_2_S convention than heterojunction design. c) Conceptual illustration of the *d*‐band and *p*‐band shifts after being optimized with different strategies.

Herein, based on the above analysis, we conduct a fundamental study to investigate how manipulating the electronic structure of polar bifunctional catalysts through heterojunction design and doping engineering determines catalytic activity. To the best of our knowledge, this study represents the first comprehensive investigation encompassing both effective strategies for optimizing polar catalysts. ZnSe, CoSe_2_/ZnSe heterostructure, as well as Co‐doped ZnSe (Co_0.125_Zn_0.875_Se), were synthesized using an in situ selenylation strategy at a mild temperature derived from MOFs (**Figure** **1**a). These materials were then used as bifunctional electrocatalysts in the modified separators for Li‐S batteries. The catalytic activity of these three catalysts has been compared, and the catalytic mechanism, as well as its relationship with intrinsic electronic structures, has also been thoroughly investigated through both experiments and theoretical analyses. Specifically, both heterojunction design and doping engineering modifications have significantly improved the deposition and decomposition behavior of Li_2_S and increased the binding energy of polysulfides on the catalysts’ surface compared to pure ZnSe. In particular, when compared to the CoSe_2_/ZnSe heterostructure, Co‐doped ZnSe exhibits a more pronounced bidirectional catalytic effect and fast anchoring of LiPSs due to the optimized electronic structure and upward shift of the *d*‐band center of metal sites and *p*‐band center of Se active sites induced by uniform Co doping (Figure 1b,c). Additionally, assisted by the incorporation of low Co^2+^ dopants, subtle lattice strain in Co_0.125_Zn_0.875_Se was obtained, leading to an increase in the exposure of active sites. Benefiting from the efficient anchoring and catalytic ability of the Co‐doped ZnSe catalyst, a substantial improvement in the electrochemical redox kinetics of Li‐S batteries has been achieved. The Li‐S cells employing the Co_0.125_Zn_0.875_Se catalyst exhibit remarkable cycling stability, maintaining a high‐capacity retention rate of 72% over 100 cycles at a low rate of 0.5 C. Exceptional rate capability (a capacity of 828 mAh g^−1^ at a high rate of 3 C), and excellent long‐cycle stability (a capacity decay of only 0.048% per cycle at 2 C over 1000 cycles) can be achieved. Even under challenging conditions with a sulfur loading of 6.6 mg cm^−2^ and a limited electrolyte volume (E/S ratio = 6 µL mg^−1^), an impressive reversible area‐specific capacity of 5.9 mAh cm^−2^ was achieved at a current rate of 0.1 C. We believe that this comprehensive study, encompassing both effective strategies for optimizing polar catalysts, provides a profound understanding of atomic engineering in the design of polar bifunctional catalysts for high‐performance Li‐S batteries.

## 2 Result and Discussion

**Figure 2:**
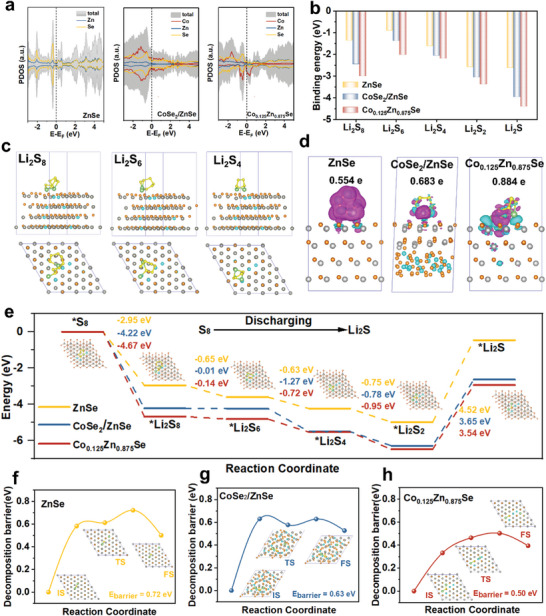
a) The calculated PDOS near the Fermi level for the ZnSe, CoSe_2_/ZnSe, and Co_0.125_Zn_0.875_Se. b) The adsorption energy between polysulfide and catalysts. c) Optimized configurations of Li_2_S_8_, Li_2_S_6_, and Li_2_S_4_ on Co_0.125_Zn_0.875_Se surfaces. d) Charge density difference for the Li_2_S_6_ on the surfaces of various catalysts. e) Gibbs energy profiles for the reduction from S_8_ to Li_2_S on ZnSe, CoSe_2_/ZnSe, and Co_0.125_Zn_0.875_Se surface. F,g) Li_2_S decomposition energy barrier on ZnSe, CoSe_2_/ZnSe, and Co_0.125_Zn_0.875_Se surfaces.

To investigate the fundamental interactions of soluble LiPSs intermediates and different catalysts at the atomic level, density functional theory (DFT) calculations were conducted to explore the electronic performance and *d*‐band center of catalysts, as well as the enhancement of binding affinities and bidirectional sulfur conversion effects on optimized polar catalyst surfaces. The optimization process involved three distinct crystal faces (111, 220, and 311) of ZnSe, along with the calculation of adsorption energies for Li_2_S_4_ on the ZnSe surface (Figure S1, Supporting Information). The outcomes indicate that the (111) surface of ZnSe exhibits slightly higher adsorption energies with Li_2_S_4_. The selection of the (111) surface for ZnSe calculations was grounded in its predominant peak observed in the standard PDF card. Three models, specifically the single ZnSe, Co_0.125_Zn_0.875_Se, and CoSe_2_/ZnSe heterointerface, were considered in the simulation (Figure S2, Supporting Information). The projected density of states (PDOS) profiles for ZnSe, CoSe_2_/ZnSe, and Co_0.125_Zn_0.875_Se are shown in **Figure** **2**a. The PDOS profiles indicate that pure ZnSe exhibits semiconductor characteristics, while CoSe_2_/ZnSe and Co_0.125_Zn_0.875_Se show the absence of a state gap at the Fermi level, demonstrating the intrinsic conductivity of the CoSe_2_/ZnSe heterojunction and Co_0.125_Zn_0.875_Se. In addition, the relative *d*‐band and *p*‐band centers of the three catalysts were investigated, revealing an adaptable band structure. Both the heterojunction design and doping engineering of ZnSe can raise the *d*‐band center (−0.71 eV in CoSe_2_/ZnSe, −0.67 eV in Co_0.125_Zn_0.875_Se) of metal atoms and *p*‐band centers (−0.40 eV in CoSe_2_/ZnSe, −0.31 eV in Co_0.125_Zn_0.875_Se) of Se active sites, conferring an inherent advantage that bolsters the interaction between polysulfides and catalysts based on the band center theory. In detail, a higher band center intensifies catalyst adsorption. Consequently, the Co_0.125_Zn_0.875_Se sample exhibits the superior anchoring capability to LiPSs compared to the CoSe_2_/ZnSe heterojunction samples. The energy band structure of Co_0.125_Zn_0.875_Se and CoSe_2_/ZnSe were performed to investigate the intrinsic conductivity of polar catalysts. As shown in Figure S3, Supporting Information, both the Co_0.125_Zn_0.875_Se and CoSe_2_/ZnSe exhibit a lack of a band gap at the Fermi level, indicating their metallic nature. The molecular architectures and binding energies of various LiPSs intermediates on the surfaces of ZnSe, CoSe_2_/ZnSe, and Co_0.125_Zn_0.875_Se are depicted in Figure 2b,c, and Table S1, and Figures S4–S6, Supporting Information. It is noteworthy that Co_0.125_Zn_0.875_Se exhibits a significantly stronger absorbability in comparison to CoSe_2_/ZnSe and pure ZnSe, which is consistent with the *d*‐band center results. Moreover, the observed electron density difference establishes a positive correlation between binding energy and electron density, as shown in Figure 2d. These analyses further substantiate the presence of substantial electron density between each Li atom in Li_2_S_6_ and its adjacent Se atom in the polar catalyst within the Li‐Se bonds, indicative of a robust covalent bonding interaction.

The Gibbs free energy profiles of the reactions from the initial S_8_ to LiPSs (Li_2_S_8_, Li_2_S_6_, and Li_2_S_4_), and then to insoluble products (Li_2_S_2_ and Li_2_S) on the surfaces of ZnSe, CoSe_2_/ZnSe, and Co_0.125_Zn_0.875_Se were investigated (Figure S7, Supporting Information). The optimized configurations of the reactive intermediates, along with their corresponding profiles of relative free energy, are shown in Figure 2e. Negative energy differences within these profiles denote spontaneous reactions, while positive energy differences indicate non‐spontaneous processes. The results demonstrate that the conversion from S_8_ to Li_2_S_8_ is exothermic. Notably, the Gibbs free energy associated with the conversion from S_8_ to Li_2_S_8_ on Co_0.125_Zn_0.875_Se and CoSe_2_/ZnSe heterojunction surface shows a higher degree of spontaneous exothermicity than that on the ZnSe surface. The final reduction step (from Li_2_S_2_ to Li_2_S) exhibits a comparatively larger positive Gibbs energy barrier than the previous steps, suggesting that the solid‐to‐solid conversion reaction process plays a pivotal role as the rate‐determining step during the discharge process. The reaction energies for the rate‐limiting step are 4.52 eV for ZnSe, 3.65 eV for CoSe_2_/ZnSe, and 3.54 eV for Co_0.125_Zn_0.875_Se, implying a heightened thermodynamic favorability for the solid‐to‐solid conversion process to occur on the Co_0.125_Zn_0.875_Se surface. To further investigate the enhanced catalytic kinetics induced by metal element doping and heterojunction structure design, the decomposition barrier of Li_2_S was also calculated. Figure 2f–h illustrates the energy barriers for the decomposition of Li_2_S on the surfaces of CoSe_2_/ZnSe (0.63 eV) and Co_0.125_Zn_0.875_Se (0.50 eV), showing a reduction in comparison to the barrier observed on ZnSe (0.72 eV) (Figures S8–S10, Supporting Information). A comprehensive comparison of two distinct polar catalysts, focusing on the electronic structure, polysulfide adsorption ability, and energy barriers associated with Li_2_S nucleation and decomposition, unequivocally affirms that the Co_0.125_Zn_0.875_Se possesses the enhanced electronic structure, superior anchoring, and lowest convention barrier energy. This finding emphatically underscores the inherent catalytic prowess of Co_0.125_Zn_0.875_Se in facilitating the charging and discharging process.

**Figure 3:**
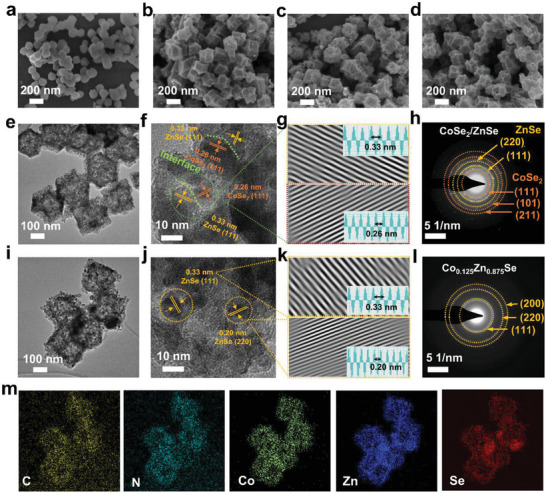
SEM images of a) Co_1_Zn_7_‐MOF, b) ZnSe, c) CoSe_2_/ZnSe, and d) Co_0.125_Zn_0.875_Se. e) TEM images of CoSe_2_/ZnSe. f) High‐resolution TEM image, g) the IFFT lattice images of the selected area (inset: Lattice distance profiles), and h) the SAED pattern of CoSe_2_/ZnSe. i) TEM images of Co_0.125_Zn_0.875_Se. j) High‐resolution TEM image, k) the IFFT lattice images of the selected area (inset: Lattice distance profiles), and l) the SAED pattern of Co_0.125_Zn_0.875_Se. m) Corresponding EDS elemental mapping of C, N, Co, Zn, and Se of the Co_0.125_Zn_0.875_Se.

The typical Zn–based compounds (ZnSe, CoSe_2_/ZnSe, and Co_0.125_Zn_0.875_Se) were synthesized by in situ selenylation of MOFs at a mild temperature. To begin with, Co*_x_*/Zn*_y_*‐MOFs regular dodecahedral particles were synthesized via self‐assembly of mental nitrate and 2‐methylimidazole in methanol solvent, as described in the previous studies with some modifications (Figure S11, Supporting Information).^[^ ^42^ ^]^ Subsequently, the obtained samples were reacted with selenium powder in a zone‐heating reactor, which underwent a one‐pot carbonization–selenylation process at a mild temperature of 600 °C to obtain the polar bifunctional electrocatalyst (Figure S12, Supporting Information). The morphology of obtained samples was characterized using advanced imaging techniques, including field‐emission scanning electron microscopy (FESEM), transmission electron microscopy (TEM), and high‐resolution field scanning electron microscopy (HRTEM). The obtained FESEM and TEM images illustrate the intricately designed porous polyhedral nanoarchitecture of catalysts, with an approximate diameter of 100 nm, as shown in **Figure** **3**a. Remarkably, ZnSe, CoSe_2_/ZnSe, and Co_0.125_Zn_0.875_Se show the same 3D morphology as the precursor (Figure 3b–d), suggesting that the carbonization and selenylation processes did not change the original morphology of the MOFs. Additionally, the observed hollow nanostructure of three catalysts primarily originates from the differential diffusivity of zinc and selenium. This hollow feature not only mitigates volume expansion during lithiation but also functions as a nano‐reactor for catalytic conversion of LiPSs. The HRTEM analysis clearly reveals the crystal structure of CoSe_2_/ZnSe heterostructure, with lattice fringes of 0.33 and 0.26 nm corresponding to the (111) crystal facets and (111) crystal facets of ZnSe and CoSe_2_, respectively (Figure 3e–g). Moreover, the selected area electron diffraction pattern (SAED) in Figure 3h exhibits diffraction rings corresponding to the (111), (101), and (211) lattice planes of CoSe_2_, affirming the excellent crystalline nature of samples. It also confirms the coexistence of CoSe_2_ and ZnSe within the carbon frameworks. The elemental mapping conducted using energy‐dispersive spectroscopy (EDS) reveals complete overlap between each element, indicating the successful dispersion of interface within the obtained heterostructure (Figure S13, Supporting Information). As shown in Figure 3i–k, highly resolved well‐defined lattice spacings of 0.33 and 0.20 nm of Co_0.125_Zn_0.875_Se were observed in the HRTEM image, corresponding to the (111) facet and (220) facet of ZnSe (Figures S14 and S15, Supporting Information), which is consistent with the results from the SAED results in Figure 3l. The elemental mapping in Figure 3m reveals a homogeneous distribution of C, N, Zn, Co, and Se elements throughout the entire Co_0.125_Zn_0.875_Se particles, which indicates the transition metal Co dopant uniformly dispersed in the ZnSe catalyst. The obtained SEM images illustrate the Co_0.05_Zn_0.95_Se and Co_0.2_Zn_0.8_Se particles with an approximate diameter of 100 nm, as shown in Figures S16 and S17, Supporting Information. The CoSe_2_ particles show the same morphology as ZnSe, as shown in Figure S18, Supporting Information. The elemental mapping reveals a homogeneous distribution of C, N, Co, and Se elements throughout the entire CoSe_2_ particles.

**Figure 4:**
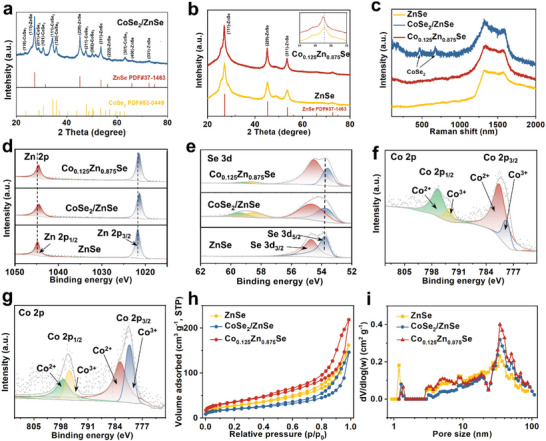
XRD patterns of a) CoSe_2_/ZnSe, and b) ZnSe and Co_0.125_Zn_0.875_Se. c) Raman spectra of ZnSe, CoSe_2_/ZnSe, and Co_0.125_Zn_0.875_Se. High‐resolution XPS spectra of the d) Zn 2p, and e) Se 3d of ZnSe, CoSe_2_/ZnSe, and Co_0.125_Zn_0.875_Se. f) High‐resolution XPS spectra of Co 2p of CoSe_2_/ZnSe. g) High‐resolution XPS spectra of Co 2p of Co_0.125_Zn_0.875_Se. h) Nitrogen adsorption–desorption isotherm and i) pore‐size distributions of ZnSe, CoSe_2_/ZnSe, and Co_0.125_Zn_0.875_Se.

The crystal structures of all samples were verified by X‐ray diffraction (XRD) characterization. The introduction of Co into Zn‐MOFs shows the same standard counterpart with Zn‐MOFs due to the closely matched atomic radius and electronegativity, confirming the successful synthesis of the bimetallic MOFs precursors (Figure S19, Supporting Information). After the selenylation process, the XRD patterns provide precise identification of ZnSe (PDF#37‐1463), both ZnSe and CoSe_2_ (PDF#53‐0449), and ZnSe (PDF#37‐1463), corresponding to the ZnSe, CoSe_2_/ZnSe heterostructure, and Co_0.125_Zn_0.875_Se, respectively. As shown in **Figure** **4**a, the successful synthesis of the heterostructures is evidenced by the presence of both CoSe_2_ and ZnSe phases in the CoSe_2_/ZnSe structures, with no additional peaks observed, indicating the absence of impurities. Notably, facilitated by the comparable ionic radii of Zn and Co ions, the successful doping of Co atoms into the ZnSe lattice is suggested by the close resemblance of diffraction peaks between the doped samples and pristine ZnSe, without the emergence of new peaks, except for a slight shift in the main peak towards the small‐angle direction (Figure 4b). This phenomenon can be attributed to the mild lattice strain effect, which is assisted by low Co^2+^ doping, inducing the exposure of active sites in Co_0.125_Zn_0.875_Se. As shown in Figure S20, Supporting Information, the different doping content of Co in ZnSe were successfully synthesized. Co_0.05_Zn_0.95_Se is evidenced by the XRD patterns, with no additional peaks observed, indicating the absence of impurities. However, with an increase in the dosage of transition metal Co ions (20%), the coexistence of augmented Zn atoms and Co atoms within the ZnSe crystal structure becomes challenging, leading to the formation of a novel cubic CoSe_2_ phase (PDF#53‐0449). As shown in Figure S21, Supporting Information, the successful synthesis of the CoSe_2_ is evidenced by the XRD patterns, with no additional peaks observed, indicating the absence of impurities. The characteristic Raman spectra of ZnSe, CoSe_2_/ZnSe heterostructure, and Co_0.125_Zn_0.875_Se in Figure 4c exhibit two well‐defined peaks at ≈1341 and 1590 cm^−1^, which can be attributed to the D and G bands of carbon species, respectively. The CoSe_2_/ZnSe (1.06) and Co_0.125_Zn_0.875_Se (1.1) exhibit a higher D/G band intensity proportion compared to ZnSe (1.03) suggesting more defect structures after optimization. It is worth noting that the Raman spectra of the CoSe_2_/ZnSe heterostructure composite show two characteristic peaks (E_g_ and A_1g_) at ≈469 and 672 cm^−1^, attributed to the presence of CoSe_2_.^[^ ^43^, ^44^, ^45^ ^]^

XPS measurements were used to investigate the valence state and electronic structure of the three catalysts. The full XPS spectra of the various samples confirm the presence of Se, Zn, C, and N elements in three ZnSe catalysts (Figure S22, Supporting Information). Additionally, Co elements can also be observed in CoSe_2_/ZnSe and Co_0.125_Zn_0.875_Se samples. The high‐resolution XPS spectra of C 1s and N 1s for all three samples are depicted in Figures S23–S25, Supporting Information. The peaks observed at 284.9, 285.9, and 289.0 eV in the C 1s spectrum are attributed to C–C, C–N, and C–O bonds, respectively. Furthermore, the fitting peaks observed at 400.9, 399.5, and 398.5 eV in the N 1s spectrum correspond to graphitic N, pyrrolic N, and pyridinic N, respectively. The N‐doped carbon structure enhances electronic and ion conductivity, as well as provides additional adsorption capacity for polysulfides.^[^ ^46^, ^47^ ^]^ As shown in Figure S26, Supporting Information, the density of state of a) C, b) graphitic N doping C, c) pyridinic N doping C, and d) pyrrolic N doping C were obtained. The results show that three forms of N doping can enhance the conductivity of C. The adsorption energy between graphitic N doping C (−0.55 eV), pyridinic N doping C (−1.5 eV), pyrrolic N doping C (−2.1 eV) for Li_2_S_6_ is larger than that of pure C (−0.54 eV), indicating that the introduction of N can enhance the chemisorption force to polysulfides. As shown in Figure 4d,e, High‐resolution XPS spectra of the Zn 2p, and Se 3d of Co_0.125_Zn_0.875_Se show no significant changes compared to the undoped ZnSe, indicating that Zn remains in the +2 oxidation state, while Se retains its −2 oxidation state. The Zn 2p and Se 3d peaks of both CoSe_2_/ZnSe and Co_0.125_Zn_0.875_Se exhibit slight negative shifts compared to those of pure ZnSe due to an electron cloud bias from the Co side to the Zn side, originating from interlayer of heterostructure or Co doping. Specifically. By substituting cations of a parent ZnSe lattice, the varying electron affinities of Co^2+^ and lattice stress resulted in increased electron density for both Zn and Se in Co‐doped ZnSe. In essence, the doping of Co in the ZnSe lattice corresponds to p‐type doping, favoring an enhancement of the material's *d*‐band and *p*‐band centers and improving its affinity for polysulfide adsorption. As shown in the high‐resolution Co 2p spectrum of CoSe_2_/ZnSe (Figure 4f), the peaks at 780.8 and 796.6 eV correspond to Co 2p_3/2_ and Co 2p_1/2_ of Co^2+^ (Figure 4f), and the peaks at 780.0 and 793.5 eV belong to Co 2p_3/2_ and Co 2p_1/2_ of Co^3+^, respectively. Similarly, the Co 2p XPS spectra of Co_0.125_Zn_0.875_Se (Figure 4g) exhibit peaks centered at 782.7 and 797.5 eV, which correspond to Co 2p_3/2_ and Co 2p_1/2_ of Co^2+^, and the peaks at 780.3 and 795.8 eV belong to Co 2p_3/2_ and Co 2p_1/2_ of Co^3+^, respectively. Notably, the Co_0.125_Zn_0.875_Se spectra display shifts in the Co 2p peaks towards higher binding energy compared to that in the CoSe_2_/ZnSe heterostructure, indicating that the electrons of the Co element have been transferred to Se and Zn, which is consistent with the XPS spectrum results of Zn 2p and Se 3d. These comprehensive analyses unequivocally confirm the successful incorporation of Co into the ZnSe lattice structures and the successful preparation of the CoSe_2_/ZnSe heterostructure. The N_2_ adsorption‐desorption analysis in Figure 4h reveals that Co_0.125_Zn_0.875_Se exhibits a higher specific surface area (127 m^2^ g^−1^) and pore volume (0.34 cm^3^ g^−1^) compared to CoSe_2_/ZnSe (69 m^2^ g^−1^, 0.23 cm^3^ g^−1^) and ZnSe (98 m^2^ g^−1^, 0.25 cm^3^ g^−1^) (Table S2, Supporting Information). This increase in specific surface area can be attributed to the preservation of the carbon skeleton from the precursor, which results in the presence of an observable microporous structure and the creation of abundant surface‐active sites (Figure S27, Supporting Information). The sites can facilitate the adsorption and transformation of polysulfide molecules. Moreover, the microporous structure effectively restrains the dissolution of polysulfides and enhances the acceleration of ion/electron transport during the electrocatalytic process, thereby contributing to improved performance (Figure 4i).^[^ ^48^ ^]^

**Figure 5:**
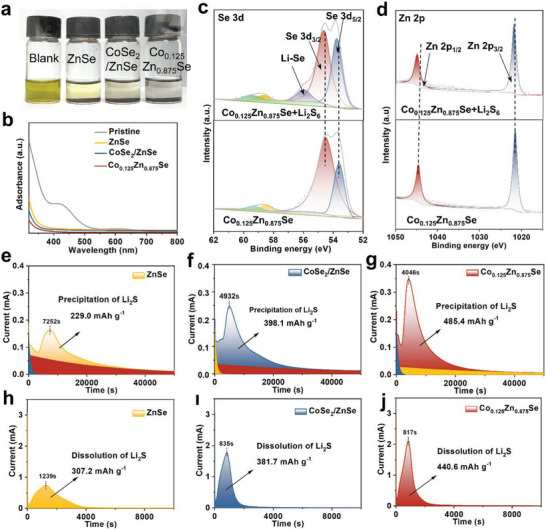
a) Optical photographs and b) corresponding UV–vis spectra of Li_2_S_6_ solutions after adsorption for blank, ZnSe, CoSe_2_/ZnSe, and Co_0.125_Zn_0.875_Se, respectively. c) Se 3d and d) Zn 2p high‐resolution XPS fine spectra of Co_0.125_Zn_0.875_Se before and after adsorption. e–g) Chronoamperometry curves of a Li_2_S_8_ solution at 2.05 V on ZnSe, CoSe_2_/ZnSe, and Co_0.125_Zn_0.875_Se electrodes. h–j) The dissolution profiles of Li_2_S.

In order to compare the adsorption capability of the ZnSe, CoSe_2_/ZnSe, and Co_0.125_Zn_0.875_Se samples toward LiPSs, static adsorption measurements by using Li_2_S_6_ solutions (10 mmol L^−1^) were conducted. As demonstrated by the optical photo (**Figures** **5**a and S28, Supporting Information), the optical photographs illustrate the color changes throughout the reaction process. The solution with Co_0.125_Zn_0.875_Se samples becomes almost no color after 3 h, while others still show light color, consistently indicating stronger chemical adsorption between the Co_0.125_Zn_0.875_Se and LiPSs. An in‐depth analysis of the chemical interactions between the catalysts and LiPSs was further carried out by using XPS. The XPS results reveal a more pronounced chemical interaction between the transition metal selenide and LiPSs, manifested by the formation of notable Se‐Li bonds. As shown in Figure 5c, the Se 3d_3/2_ and Se 3d_5/2_ peaks of Co_0.125_Zn_0.875_Se shift to higher values after Li_2_S_6_ absorption, and a new peak at 55.5 eV emerge, indicating the presence of Li‐Se bonds. This finding is corroborated by the corresponding differential charge distribution and adsorption results in DFT calculations, demonstrating a substantial charge transfer from Co atoms to Zn and Se atoms within the Co_0.125_Zn_0.875_Se. Furthermore, the fitted high‐resolution XPS curves of Co_0.125_Zn_0.875_Se exhibit shifts in the Zn 2p_1/2_ and Zn 2p_3/2_ peaks from ≈1021.4 and 1044.5 to 1021.7 and 1044.8 eV, respectively, after the adsorption of Li_2_S_6_ (Figure 5d). the fitted high‐resolution XPS curves of Zn 2p in CoSe_2_/ZnSe also show the same trend (Figure S29, Supporting Information). Similarly, the Co 2p_3/2_ and 2p_1/2_ peaks of LiPSs‐treated Co_0.125_Zn_0.875_Se and CoSe_2_/ZnSe display higher binding energies (Figure S30, Supporting Information). These shifts in peak positions can be attributed to interactions between active sites in catalysts and sulfur species.^[^ ^42^ ^]^

To establish a quantitative correlation between different optimization strategies and electrocatalytic performance, a series of electrochemical tests were conducted. In the charge and discharge process of Li‐S batteries, the electrode reaction involves liquid‐liquid, liquid‐solid, and solid‐liquid conversion. The catalytic effect of Co_0.125_Zn_0.875_Se and CoSe_2_/ZnSe heterostructure on Li_2_S precipitation in the liquid‐solid conversion reaction during discharging were investigated through the evaluation of Li_2_S nucleation and growth, as shown in Figure 5e–g. Among the three samples, Co_0.125_Zn_0.875_Se exhibits the most rapid response in Li_2_S nucleation, surpassing CoSe_2_/ZnSe heterostructure and pure ZnSe. Furthermore, the capacity of Li_2_S precipitation on Co_0.125_Zn_0.875_Se (485.4 mAh g^−1^) surface exceeds that on CoSe_2_/ZnSe (398.1 mAh g^−1^), and ZnSe (229.0 mAh g^−1^) surface (Figure S31, Supporting Information). The SEM was utilized to examine the morphologies of Li_2_S deposited on various catalyst supports. A uniform Li_2_S deposition was observed on the Co_0.125_Zn_0.875_Se surface, whereas Li_2_S aggregation occurred on the part of the CoSe_2_/ZnSe surface (Figure S32, Supporting Information). These findings unequivocally demonstrate the significant reduction of overpotential for the initial nucleation of Li_2_S and the promotion of Li_2_S precipitation by Co_0.125_Zn_0.875_Se. The process of sulfur evolution reaction (SER) also encounters a significant challenge due to the high dissociation energy barrier of Li_2_S, hindering its efficient decomposition. Hence, achieving optimal Li_2_S manipulation requires a combination of robust LiPS reduction and efficient Li_2_S dissociation. The dissolution of Li_2_S was investigated by potentiostatic discharge/charge experiments to show the enhanced solid‐liquid conversions. Co_0.125_Zn_0.875_Se in Figure 5j presents a larger dissolution capacity (440.6 mAh g^−1^) and faster conversion time (817 s) for transforming solid Li_2_S into soluble LiPSs, surpassing CoSe_2_/ZnSe in Figure 5i (381.7 mAh g^−1^, 835 s) and ZnSe in Figure 5h (307.2 mAh g^−1^, 1239 s). The remarkable nucleation and dissolution capacity of Li_2_S observed in Co_0.125_Zn_0.875_Se suggests its effective catalysis of the liquid‐solid and solid‐liquid conversion kinetics between LiPSs and Li_2_S.^[^ ^49^ ^]^

**Figure 6:**
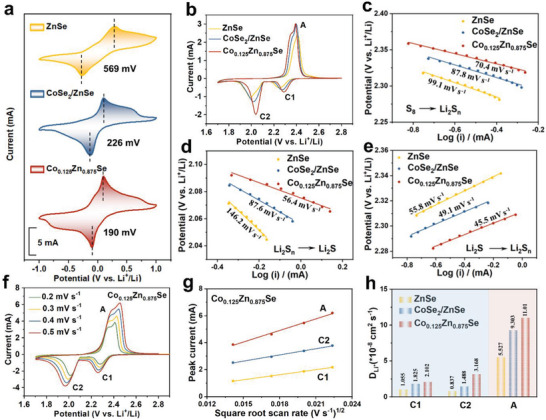
a) CV curves of the symmetrical cells with ZnSe, CoSe_2_/ZnSe, and Co_0.125_Zn_0.875_Se electrodes in electrolytes with Li_2_S_6_. b) CV curves of ZnSe, CoSe_2_/ZnSe, and Co_0.125_Zn_0.875_Se, and its corresponding Tafel slopes in c) C1 (S_8_→Li_2_S_n_), d) C2 (Li_2_S_n_→Li_2_S), and e) A (Li_2_S → Li_2_S_n_). f) CV curves of Co_0.125_Zn_0.875_Se‐based cell at different scan rates. g) The Li‐ion diffusion properties of a Co_0.125_Zn_0.875_Se‐based cell investigated by analyzing the CV peak currents for peaks C1, C2, and A in relation to the square root of the scan rates. h) The diffusion coefficient of Li‐ion calculated from the CV redox peaks according to the Randles–Sevcik equation.

Symmetric cells with Li_2_S_6_‐containing electrolytes were assembled to evaluate the electrocatalytic activities during the liquid‐liquid conversion process. Remarkably, when Co_0.125_Zn_0.875_Se was used as the electrode material, redox peaks show smallest voltage hysteresis (190 mV) and sharp redox peaks with the highest current responses (**Figures** **6**a and S33, Supporting Information), indicating that Co_0.125_Zn_0.875_Se exhibits a remarkable ability to enhance the electrochemical reaction kinetics of polysulfides in comparison with CoSe_2_/ZnSe. The overall accelerated conversions in the liquid‐liquid, liquid‐solid, and solid‐liquid phases further support the excellent bidirectional catalytic activity of the doping engineering strategy in facilitating LiPS redox kinetics. This effect can be attributed to the higher *d*‐band center and electrode transfer due to the dope element in the polar catalyst, which reduces the energy barrier of the decomposition reaction and promotes the conversion of LiPSs. Hence, the strategy of doping engineering in polar catalysts plays a crucial role in enhancing the electrocatalytic performance compared to heterojunction design.

The cyclic voltammetry (CV) curves exhibit two distinctive cathodic peaks at ≈2.3 and 2.0 V, corresponding to the reduction of S_8_ to initiate the formation of long‐chain LiPSs, followed by their subsequent conversion to short‐chain Li_2_S_2_/Li_2_S (Figure 6b). An oxidation peak observed around 2.4 V during the anodic sweep can be attributed to the transitions from Li_2_S_2_/Li_2_S back to S_8_. A representation of the precise peak positions for each reaction, as influenced by the various catalysts, is visually illustrated. Notably, the peak potentials exhibits in the reduction processes (C1 and C2) demonstrate a clear increase in Li‐S batteries incorporating Co_0.125_Zn_0.875_Se and CoSe_2_/ZnSe catalysts. In contrast, the peak potentials during the oxidation process (A) manifest a notable decrease in these batteries, which signifies a significant reduction in polarization after optimization, particularly the doping engineering. This reduction in polarization is indicative of the highly promoted conversion of LiPSs facilitated by the Co_0.125_Zn_0.875_Se catalyst during the redox reactions. In order to further evaluate the electrocatalytic effect, Tafel curves derived from the corresponding redox peaks in the CV profiles are meticulously plotted, as shown in Figure 6c‐e. The fitted Tafel slopes from S_8_ to Li_2_S_n_ are determined to be 99.1, 87.8, and 70.4 mV dec^−1^ for Li‐S batteries employing the ZnSe, CoSe_2_/ZnSe, and Co_0.125_Zn_0.875_Se as catalysts, respectively. Additionally, the slopes from Li_2_S*_n_* to Li_2_S_2_/Li_2_S are measured to be 146.2, 87.6, and 56.4 mV dec^−1^ for Li‐S batteries with ZnSe, CoSe_2_/ZnSe, and Co_0.125_Zn_0.875_Se catalysts, respectively. Further analysis of the Tafel slopes during the oxidation processes also shows that the Co_0.125_Zn_0.875_Se exhibits the smallest slope values. The decrease in the Tafel slopes for Co_0.125_Zn_0.875_Se suggests a mitigated solid‐liquid‐solid redox barrier and accelerated LiPSs conversion kinetics, compared to pure ZnSe and the CoSe_2_/ZnSe heterostructure.

The Li‐ion diffusion coefficient serves as an insightful descriptor to determine the electrocatalytic impact of diverse catalysts during the redox reactions of sulfur species. CV profiles collected at scan rates ranging from 0.2 to 0.5 mV s^−1^ enable the investigation of the Li‐ion diffusion coefficient (Figure 6f, and Figure S34, Supporting Information). A linear fitting model is employed to describe the relationship between the peak current and the square root of the scan rate, which suggests a diffusion‐limited process. The Randles–Sevcik equation is utilized to capture the dynamics of Li‐ion diffusion. Notably, steeper fitted curves observed for Co_0.125_Zn_0.875_Se are indicative of significantly higher Li‐ion diffusion coefficients relative to those of CoSe_2_/ZnSe throughout each reaction process (Figure 6g,h). The GITT experiments were studied to investigate the diffusion coefficient of lithium ions in different catalysts, as shown in Figure S35, Supporting Information. These results reinforce the notion of notably faster diffusion and reaction kinetics on the Co_0.125_Zn_0.875_Se surface, further validating the excellent electrocatalytic effect of redox reaction facilitated by the robust Co_0.125_Zn_0.875_Se catalyst. In conclusion, based on various catalytic performance testing experiments, the Li‐S batteries with CoSe_2_/ZnSe heterostructure achieve higher electrocatalytic effect than the pristine ZnSe but fall short compared to the Co_0.125_Zn_0.875_Se. This difference can be attributed to the partial mitigation of the LiPSs shuttling effect by the CoSe_2_/ZnSe heterostructure. However, it remains fundamentally unable to prevent the dissolution of LiPSs due to weaker interactions and slower kinetics, as supported by DFT calculations.

**Figure 7:**
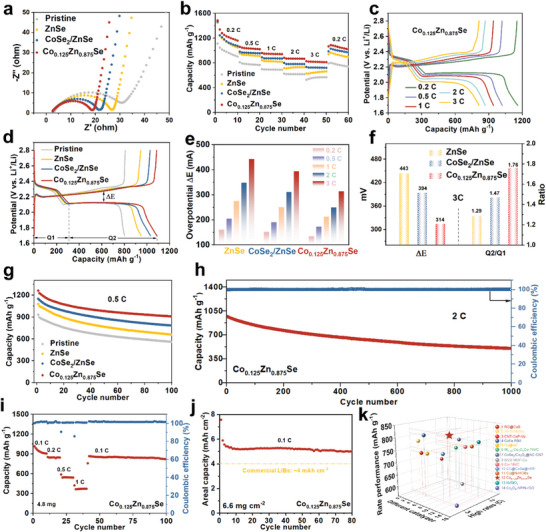
a) Electrochemical impedance spectroscopy (EIS) spectra of ZnSe, CoSe_2_/ZnSe, and Co_0.125_Zn_0.875_Se. b) Rate performance of ZnSe, CoSe_2_/ZnSe, and Co_0.125_Zn_0.875_Se. c) Galvanostatic charge/discharge profiles with different current rates of Co_0.125_Zn_0.875_Se. d) Charge/discharge profiles at different catalysts at 0.2C after cycling. e) ΔE values obtained from galvanostatic charge–discharge curves of various catalysts. f) ΔE and Q2/Q1 values obtained from galvanostatic charge–discharge curves of various catalysts at current density 3 C. g) Cycling performance at 0.5 C. h) Long‐term cycle performance at 2 C of Li‐S cells with Co_0.125_Zn_0.875_Se. i) Rate performance of Co_0.125_Zn_0.875_Se at 4.8 mg cm^−2^. j) Cycling performance of Co_0.125_Zn_0.875_Se at 6.6 mg cm^−2^. k) Comparison of the electrochemical performance between Co_0.125_Zn_0.875_Se‐based cells and other catalysts for Li‐S batteries.

Many experiments were conducted to evaluate the enhanced cathode performance of cells through different catalysts. Sulfur was infiltrated into commercially available CNT using a standard melt diffusion technique, yielding a C/S composite containing 70 wt% sulfur content. EIS tests were first measured to compare the charge‐transfer resistance through Nyquist plots. Notably, the Co_0.125_Zn_0.875_Se electrode exhibits the lowest charge‐transfer resistance, indicating the fastest charge transfer at the catalyst/LiPSs interface (**Figure** **7**a). The electrochemical cycling stability and rate performance of cells with different catalyst‐based modified separators were investigated. The rate performance of cells with various catalysts is evaluated, as illustrated in Figure 7b. The Co_0.125_Zn_0.875_Se‐based cells display the highest rate‐charge and discharge performance. It exhibits specific capacities of 1486, 1059, 960, 881, and 828 mAh g^−1^ at 0.2, 0.5, 1, 2, and 3 C, respectively. In contrast, cells using CoSe_2_/ZnSe‐based and ZnSe‐based separators show lower capacities. Notably, the rate performance notably differed, especially at a current density of 3 C, where the Co_0.125_Zn_0.875_Se‐based cell shows a typical two‐plateau discharge curve with low voltage polarization due to moderate adsorption and optimal catalytic activities (Figure 7c–e). On the other hand, the other electrodes exhibit large voltage polarizations at the second discharge plateau (Figure S36, Supporting Information). A higher Q2/Q1 ratio indicates a proficient transformation into Li_2_S, indicating an enhanced catalytic efficiency of the catalyst. Notably, the Co_0.125_Zn_0.875_Se exhibits a remarkable Q2/Q1 value of 1.76, significantly surpassing the Q2/Q1 values observed in CoSe_2_/ZnSe and ZnSe. Moreover, the discharge‐charge profiles further reveal the capacity advantage and minimized polarization (∆E), affirming the superior catalytic ability of Co_0.125_Zn_0.875_Se (Figure 7f, and Figure S37, Supporting Information). Li‐S cells with Co_0.125_Zn_0.875_Se also exhibit exceptional capacity and cycling performance at a current density of 0.5 C. After 100 cycles, an impressive retention rate of 72% was achieved (initial capacity of 1261.3 mAh g^−1^). Notably, this performance was superior to cells with CoSe_2_/ZnSe (initial capacities of 1149.5 mAh g^−1^ with 68% retention) and pure ZnSe catalysts (initial capacities of 1078.6 mAh g^−1^ with 61% retention), or without polar catalyst (initial capacities of 931.3 mAh g^−1^ with 60% retention), as shown in Figure 7g. The capacity and cycling performance of Li‐S cells with CoSe_2_ was also tested at a current density of 0.5 C. An initial capacity of 956.8 mAh g^−1^ and a discharging capacity of 711.0 mAh g^−1^ after 50 cycles was achieved (Figure S38, Supporting Information). The capacity and cycling performance of ZnSe with different Co doping concentrations‐based Li‐S cells were also tested at a current density of 0.5 C. As shown in Figure S39, Supporting Information, the electrochemical performance demonstrates an increasing trend with the augmentation of the doping concentration. However, when the doping concentration is too high leading to the emergence of impurity peaks, the coexistence of Zn atoms and Co atoms within the ZnSe crystal lattice becomes unattainable. At this point, the electrochemical performance experiences a decline. The Co_0.125_Zn_0.875_Se‐based cell demonstrates impressive cycling stability at a high current density of 2 C, with a reversible capacity of 503 mAh g^−1^ even after 1000 cycles and a decay rate of 0.048% per cycle (Figure 7h). Moreover, the average Coulombic efficiency is over 99%, highlighting the priority of the doping strategy. Additionally, cells with high sulfur loading were fabricated to evaluate the feasibility of the practical application of catalysts. As depicted in Figure 7i, the Co_0.125_Zn_0.875_Se‐based cell, with a sulfur loading of 4.8 mg cm^−2^, exhibits impressive specific capacities of 1010, 853, 590, and 423 mAh g^−1^ at 0.1, 0.2, 0.5, and 1 C, respectively. Even with a sulfur loading of 6.6 mg cm^−2^, the long‐term cycling stability of the Co_0.125_Zn_0.875_Se‐based cell maintains a high specific capacity of 5.0 mAh cm^−2^ after more than 80 cycles at 0.1 C (Figure 7j). These electrochemical results are comparable to recently reported works (Figure 7k, and Table S4, Supporting Information).

SEM and TEM tests were used to study the morphology and crystal structure of Co_0.125_Zn_0.875_Se before and after cycling. As illustrated in Figure S40, Supporting Information, the pristine membrane modification layer reveals distinct particles of Co_0.125_Zn_0.875_Se before cycling, despite the presence of partially integrated conductive carbon and binders. Upon cycling, the SEM images of the modified membrane layer exhibit a discernible layer of lithium sulfide discharge product on the surface. This lithium sulfide originates from dissolved polysulfides in the electrolyte, indicating the effective hindrance of dissolved polysulfides from shuttling to the lithium electrode by the modified membrane layer. It is noteworthy that lithium sulfide is susceptible to high‐energy electron beams, leading to its disappearance and decomposition, a natural property of lithium sulfide. The inherent Co_0.125_Zn_0.875_Se‐based modification layer remains evident, and the morphology of nanoscale particles is the same as before cycling. TEM analysis was used to study the crystal structure of Co_0.125_Zn_0.875_Se after cycling. The results reveal an unchanged crystal structure, with diffraction rings identical to those observed before cycling, underscoring the stability of the catalyst.

**Figure 8:**
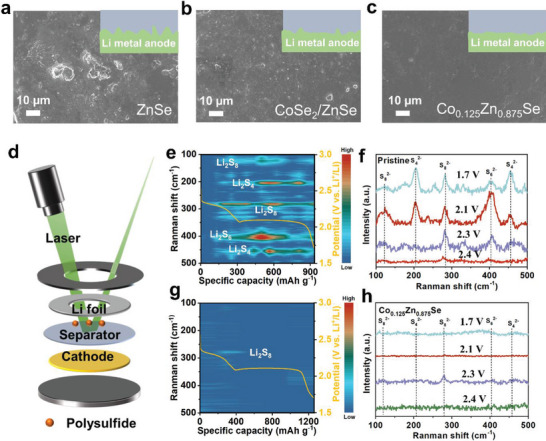
a–c) SEM images of different interlayers corresponding to lithium anodes after 100 cycles at 0.5 C. d) The setup used for in situ Raman spectroscopy analyses of the cell configuration. In situ Raman spectra of Li‐S cells using e,f) blank separator, and g,h) Co_0.125_Zn_0.875_Se‐modified separator.

As the stability of cycling performance is closely linked to the growth of lithium dendrites on its anode in Li‐S batteries, the surface structure of the lithium anode after 100 cycles at 0.5 C was analyzed by SEM. The SEM images corresponding to the ZnSe interlayers in **Figure** **8**a show that the lithium surface exhibits the most substantial corrosion. The SEI layer became uneven after cycling, accompanied by the formation of numerous moss‐like structures. Interestingly, the lithium surface of the cell based on CoSe_2_/ZnSe catalyst displays only minor corrosion (Figure 8b). Particularly noteworthy is the lithium surface of the cell using the Co_0.125_Zn_0.875_Se‐based separator (Figure 8c), which exhibits a dense and smooth morphology. This indicates minimal corrosion due to the establishment of a stable SEI layer. These findings provide strong evidence supporting the effectiveness of a rational‐designed intercalation strategy in mitigating the polysulfide shuttle effect, leading to the formation of a stable SEI layer and a reduction in the occurrence of lithium dendrite formation.

To explore the inhibition of the shuttle effect of various LiPSs during discharge processes and validate the benefits of doping engineering of polar catalysts in Li‐S batteries, in situ Raman spectra were utilized (Figure 8d). It is worth mentioning that distinct signals of long‐chain and mid‐chain LiPSs were observed during the discharge process in Li‐S cells with blank PP separators (Figure 8e,f). At the beginning of discharge, the peaks around 121, and 279 cm^−1^ were attributed to the presence of S_8_^2−^ signals. As the cell discharged at 2.3 and 2.1 V, peaks around 450 (S_4_^2−^), 405 (S_6_^2−^), and 202 cm^−1^ (S_4_^2−^) emerged. Further discharge led to a gradual reduction in the intensity of peaks associated with S_6_^2−^/S_4_^2−^, indicating the conversion of long‐chain and mid‐chain polysulfides into short‐chain polysulfides. At the end of discharge (1.7 V), a substantial amount of S_8_^2−^/S_6_^2−^/S_4_^2−^ species still remained present in the electrolyte, implying a severe shuttle effect. In contrast, the signals corresponding to LiPSs were notably weakened throughout the discharging process of the Li‐S cell with a Co_0.125_Zn_0.875_Se‐based separator (Figure 8g,h). The above findings confirm that doping engineering of polar catalysts effectively adsorbs LiPSs and facilitates their electrochemical conversions. Consequently, the shuttle effect can be effectively inhibited, and the utilization of sulfur is greatly enhanced.

## 3 Conclusion

In summary, we comprehensively explored and compared the catalytic and immobilization capabilities of LiPSs through two strategies: heterojunction design and doping engineering, based on underperforming polar ZnSe catalysts. Pure ZnSe, CoSe_2_/ZnSe heterostructure, and Co_0.125_Zn_0.875_Se were successfully fabricated via a simple method involving hydrothermal and calcination processes. These synthesized materials were employed as bifunctional electrocatalysts in modified separators. Both experiments and theoretical calculations reveal that the Co‐doping strategy enhances the chemical interaction with LiPSs, enabling robust anchoring efficacy. Simultaneously, it promotes the promotion of Li^+^ diffusion and reduces the reaction energy barrier, collectively contributing to the acceleration of redox kinetics in the liquid‐phase conversions of LiPSs and the nucleation process of Li_2_S. These improvements can be attributed to the progressive migration of the *d*‐band center and the optimized electronic conductivity. As a result, the superior Co_0.125_Zn_0.875_Se catalyst enables Li‐S batteries to achieve an outstanding discharge‐specific capacity of 1261.3 mAh g^−1^ at 0.5 C and a stable cycling performance with a decay rate of 0.048% per cycling within 1000 cycling under a high rate of 2 C. Significantly, even as the sulfur loading escalates to 6.6 mg cm^−2^, an areal capacity of 7.6 mAh cm^−2^ at 0.05 C was achieved in the first cycle, and an areal capacity of 5.9 mAh cm^−2^ at 0.1 C was obtained, steadfastly maintaining performance at 5.0 mAh cm^−2^ over 80 cycles. Raman spectroscopy results further substantiate the amelioration of electrochemical conversions of LiPSs facilitated by the presence of doping elements. This systematic investigation comprehensively explores and contrasts the systematic manipulation of the electronic structure of polar bifunctional catalysts through the strategies of heterojunction design and doping engineering, offering novel insights into the perspective of polar catalyst optimization, thus advancing the performance of Li‐S batteries.

## 4 Experimental Section

Detailed experimental procedures can be found in the Supporting Information.

## Conflict of Interest

The authors declare no conflict of interest.

## Author Contributions

H.X., Q.J., and Z.S. contributed equally to this work.

## Supporting information

Supporting Information

## Data Availability

The data that support the findings of this study are available from the corresponding author upon reasonable request.
