# Supplementary material for: Fundamentally Manipulating the Electronic Structure of Polar Bifunctional Catalysts for Lithium‐Sulfur Batteries: Heterojunction Design versus Doping Engineering
Source: Adv Sci (Weinh). 2024 Mar 11;11(20):2307995. doi: 10.1002/advs.202307995 (PMC11132031; doi:10.1002/advs.202307995)
Supplement: Supplementary file 1 — Supporting Information [file ADVS-11-2307995-s001.pdf]

ADVANCED SCIENCE
Open Access

Supporting Information

for *Adv. Sci.*, DOI 10.1002/advs.202307995

Fundamentally Manipulating the Electronic Structure of Polar Bifunctional Catalysts for Lithium-Sulfur Batteries: Heterojunction Design versus Doping Engineering

Huifang Xu, Qingbin Jiang, Zheng Shu, Kwan San Hui, Shuo Wang, Yunshan Zheng, Xiaolu Liu, Huixian Xie, Weng-Fai (Andy) Ip, Chenyang Zha, Yongqing Cai* and Kwun Nam Hui**

WILEY-VCH

Supporting Information

Fundamentally Manipulating the Electronic Structure of Polar Bifunctional Catalysts for Lithium-Sulfur Batteries: Heterojunction Design Versus Doping Engineering

Huifang Xu, Qingbin Jiang, Zheng Shu, Kwan San Hui, Shuo Wang, Yunshan Zheng, Xiaolu Liu, Huixian Xie, Weng-Fai (Andy) Ip, Chenyang Zha, Yongqing Cai,* Kwun Nam Hui**

Experimental Section

Fabrication of bimetallic Zn/Co-MOF precursor. The chemicals were used as they were received, without any further purification steps. The synthesis process is shown in Figure 1c and Supporting Information S1. $Co(NO_3)_2{\cdot}6H_2O$ (1 mmol) and $Zn(NO_3)_2{\cdot}6H_2O$ (7 mmol) with a molar ratio of 1:7 were first dissolved in methanol under magnetic stirring at room temperature for 5 mins. The aqueous methanol solution of 2-methylimidazole (90 mL, 3.94g) (Sigma-Aldrich) was quickly added to the former mixed solution under magnetic stirring. After a 6 h reaction at room temperature, the collected precipitates were washed with methanol several times, and finally dried under vacuum at 60 °C overnight to obtain the Co_1Zn_7-MOFs sample.[1] The Zn-MOFs was also prepared by the similar procedure, except without adding the $Co(NO_3)_2{\cdot}6H_2O$. The Co-MOFs was also prepared by the similar procedure, except without adding the $Zn(NO_3)_2{\cdot}6H_2O$. The Co_1Zn_1-MOF was also prepared by the similar procedure, except with a molar ratio of 1:1 ($Co(NO_3)_2{\cdot}6H_2O$ (4 mmol) : $Zn(NO_3)_2{\cdot}6H_2O$ (4 mmol)). The Co_1Zn_{19}-MOF was also prepared by the similar procedure, except with a molar ratio of 1:19 ($Co(NO_3)_2{\cdot}6H_2O$ (0.4 mmol) : $Zn(NO_3)_2{\cdot}6H_2O$ (19.6 mmol)). The Co_1Zn_4-MOF was also prepared by the similar procedure, except with a molar ratio of 1:4 ($Co(NO_3)_2{\cdot}6H_2O$ (1.6 mmol) : $Zn(NO_3)_2{\cdot}6H_2O$ (6.4 mmol)).

Fabrication of the $Co_{0.125}Zn_{0.875}Se$ materials. Typically, 200 mg as-prepared Co_1Zn_7-MOF and 400 mg Se powder were separately put in two porcelain boats, with the Se powder placed upstream and the MOFs downstream. This calcination process was carried out at 350 °C for 1 h and then at 600 °C for 2 h under an N_2 atmosphere, with a heating rate of 2 °C min^{-1}. ZnSe, $CoSe_2$, $Co_{0.05}Zn_{0.95}Se$, $Co_{0.2}Zn_{0.8}Se$ and $CoSe_2$/ZnSe heterostructure were also prepared according to the same process by using Zn-ZIF, Co-ZIF, Co_1Zn_{19}-MOF, Co_1Zn_4-MOF, and Co_1Zn_1-MOF to replace Co_1Zn_7-MOF, respectively.

Fabrication of sulfur cathode material. To synthesize the sulfur cathode materials, a blend of commercial super-p and sublimed sulfur powder at a mass ratio of 3:7 was meticulously prepared. This mixture was then transferred into an autoclave that had been filled with an Ar atmosphere. Subsequently, the autoclave was heated to 155 °C for 12 h, ultimately yielding the desired materials.

Fabrication of modified separators. Using the blade coating method, the modified separators were obtained, with the $Co_{0.125}Zn_{0.875}Se$-modified separator as an example. A slurry containing $Co_{0.125}Zn_{0.875}Se$ powder, super-p, and polyvinylidene fluoride (PVDF) in a mass ratio of 7:2:1 was coated on the Celgard PP separator and dried overnight in a vacuum oven at 60 °C for 12 h. The ZnSe, $CoSe_2$/ZnSe, and $Co_{0.125}Zn_{0.875}Se$ had an area loading of approximately 0.27 mg cm^{-2}.

Characterization of materials

The morphology of the synthesized samples underwent comprehensive analysis using a suite of advanced techniques. Field emission scanning electron microscope (FESEM, JEOL JSM-7500FA) was employed to obtain scanning electron microscope (SEM) images. Transmission electron microscopy (TEM), HRTEM images, and the corresponding EDS elemental mappings were captured using a JEOL JEM-2100 electron microscope operating at 200 kV. Powder X-ray diffraction (PXRD) patterns were obtained using a Rigaku Smartlab 9000W diffractometer with Cu Kα radiation (λ = 0.15418 nm) operating at 40 KV and 200 mA. Raman analysis was conducted on a Micro Raman System (Horiba LABHRev-UV) with 633 nm incident radiation. The specific surface area and pore size distribution were determined by BET and Barrett-Joyner-Halenda (BJH) methods, based on N_2 adsorption desorption isotherms. These isotherms were obtained at liquid nitrogen temperature (-196 °C) using Micromeritics ASAP 2020. X-ray photoelectron spectroscopy (XPS) patterns were collected using a Thermo ESCALAB 250 spectrometer with monochromatic Al Kα as the excitation

source. The C/S sample underwent TGA (NETZSCH TG 209 F3) under an Ar atmosphere with a heating rate of 10 ºC min^{-1}, starting from room temperature to 700 ºC. UV–vis spectra were obtained by a UV-Vis Spectrophotometer (Jasco V-770) (UV-Vis_Jasco).

Adsorption and catalytic studies of $CoSe_2$/ZnSe and $Co_{0.125}Zn_{0.875}Se$

The Li_2S_6 solution was prepared by combining Li_2S (Sigma-Aldrich) and sublimed sulfur (Sigma-Aldrich) in a 1:5 mass ratio within a conventional electrolyte solution, comprising 1.0 M LiTFSI dissolved in a 1:1 volume-to-volume mixture of 1,3-dioxolane and dimethyl ether, supplemented with 1.0 wt% $LiNO_3$. This resulting mixture underwent vigorous magnetic stirring at 70 ºC over an entire night, yielding a brownish-red Li_2S_6 electrolyte solution with a concentration of 1 M. To assess the adsorption capacity of LiPSs, 20 mg of functional materials were immersed in 5 ml of 2 mM Li_2S_6 solutions at room temperature.

Li_2S nucleation and decomposition measurement. To prepare a Li_2S_8 electrolyte with a concentration of 0.20 mol L^{-1}, sublimed sulfur, and Li_2S were combined in a molar ratio of 7:1, using tetraglyme as the solvent. The mixture underwent vigorous stirring under an argon atmosphere at 60 °C for 24 h. For the assembly of the coin cell, a commercial CP served as the current collector, and functional materials were dispersed as the cathode at a loading density of 0.5 mg cm^{-2}, with lithium foils employed as counter electrodes. In the cathode compartment, 25 μL Li_2S_8 electrolyte was applied onto the CP, while the anode received a traditional electrolyte without Li_2S_8 (20 μL). To induce Li_2S nucleation, the cells were discharged galvanostatically at 0.112 mA until reaching 2.06 V, followed by a potentiostatic discharge at 2.05 V until the current dropped below 10^{-5} A. Conversely, for Li_2S decomposition, the cells underwent galvanostatic discharge down to 1.70 V at a rate of 0.112 mA, and then potentiostatic charging at 2.35 V was continued until the charge current reached less than 10^{-5} A.

Assembly and measurement of symmetric cells. To create a symmetrical battery electrode, a 12 mm disc was cut from carbon paper, onto which an ethanol dispersion of ZnSe, $CoSe_2$/ZnSe, and $Co_{0.125}Zn_{0.875}Se$ particles was deposited with a loading of 0.5 mg cm^{-2}. After the deposition had dried, two identical electrodes were integrated into a standard 2023-coin cell. An electrolyte consisting of 40.0 μL Li_2S_6 was then added to the cell. To assess the performance of the symmetrical battery, cyclic voltammetry measurements were conducted using the Bio-Logic EC-LAB (VMP-300) equipment. The scan rate was set at 0.1 mV s^{-1}, and the voltage range covered -1 V to 1 V during the scans.

In situ Raman spectroscopy. The cells were assembled into an in situ Raman device with a quartz window from Beijing Scistar Technology Co. Ltd. To detect the dissolved LiPSs in the electrolyte, small holes of 2 millimeters were manufactured in the lithium film, allowing the light source to pass through. The Raman raster was set to 2400 mm^{-1}, and the wavelength used was 633 nanometers. During measurement, the cell was discharged at a current of 0.5 C, and the recorded Raman shift spanned from 100 to 500 cm^{-1}.

Electrochemical measurements of Li-S cells

To perform electrochemical tests, coin-type half-cells (2032) were meticulously assembled within an argon-filled glove box. The cathode material C/S was blended with super-p and PVDF in an 8:1:1 ratio, using a moderate amount of NMP. This mixture was stirred to achieve a uniform slurry, which was subsequently coated onto an aluminum foil and dried at 60 °C for 12 h. For the half-cells and cyclic voltammetry (CV) tests, the total mass loading was approximately 1.2 mg cm^{-2}, and the electrolyte-to-sulfur ratio was roughly 18 μL mg^{-1} for the standard coin cell configuration. These cells were constructed with lithium metal as the anode, Celgard 2500 as the separator, and the electrolyte consisted of lithium bis (trifluoromethanesulfonyl) imide (LiTFSI,

1.0 M) dissolved in a mixture of 1,3-dioxolane and 1,2-dimethoxyethane (V/V=1:1), with the addition of 1 wt% $LiNO_3$. The cycling performance and rate capabilities were evaluated using a Neware Battery Tester, within a voltage window of 1.7 V to 2.8 V, while CV tests were conducted using Bio-Logic EC-LAB (VMP-300) equipment at room temperature.

Theoretical calculations

All spin-polarized DFT calculations were performed by the projector-augmented wave (PAW) method using the Vienna ab initio Simulation Package (version 5.4.4).[2] The Perdew–Burke–Ernzerhof (PBE) form of the generalized gradient approximation (GGA) was adopted to describe the exchange-correlation functional.[3] The van der Waals interactions between substrate and adsorbate were considered by the DFT-D3 method.[4] The kinetic cut-off energy was set to 500 eV and a 2 × 2 × 1 k-points mesh was used for the first Brillouin sampling. The Gaussian smearing of 0.05 eV is used to calculate the electronic occupation. All structures were fully relaxed by the conjugate-gradient algorithm until the total energy and Hellmann-Feynman force are less than 1×10^{-5} eV and 0.05 eV $Å^{-1}$, respectively. The surface of ZnSe (111) with four atom layers was modeled by a 3 × 3 × 1 supercell, whereas a heterojunction of ZnSe (111) and $CoSe_2$ (111) is built. For the calculations of pristine and Co-doped ZnSe surfaces, only the two upper layers were allowed to relax while the two bottom layers were fixed during geometry optimization.[5] For the calculations of the heterojunction, all layers were allowed to relax. A vacuum layer of ~15 Å is added to avoid the interlayer interaction of periodic unit cells in the z-axis. The adsorption of substrate and adsorbate can be calculated by Eads = $E_{sub+adsorbate}$ - E_{sub} - $E_{adsorbate}$, where $E_{sub+adsorbate}$, E_{sub} and $E_{adsorbate}$ are the energies of the total systems, substrate, and adsorbate, respectively. Bader charge method was carried out to study the charge transfer between Li_2S_6 and substrate.[6] The differential charge density $\Delta\rho(r)$ were used to visualize the charge

transfer, which is defined as $\Delta\rho(r) = \rho_{sub+adsorbate}(r) - \rho_{sub}(r) - \rho_{adsorbate}(r)$, where $\rho_{sub+adsorbate}(r)$, $\rho_{sub}(r)$ and $\rho_{adsorbate}(r)$ are the charge densities of the total systems, substrate, and adsorbate, respectively. To figure out the diffusion energy barriers of Li_2S dissociation, climbed nudged elastic band (CI-NEB) method was performed by the quick-min algorithm.[7] All geometric structures were visualized using the VESTA package.[8]

Supplementary Figures and Tables

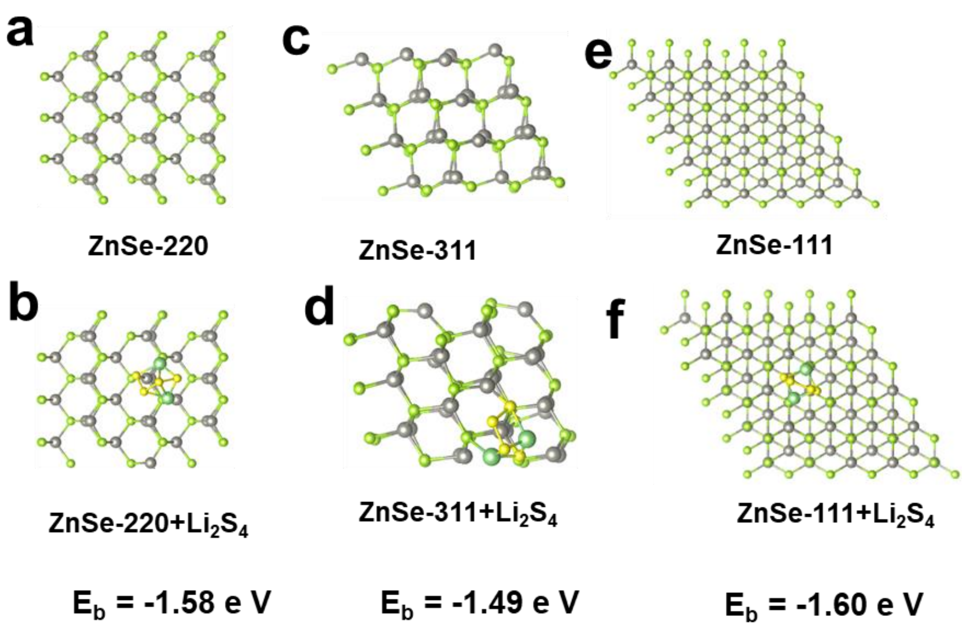

Figure S1. The optimized structural configurations of ZnSe with different surface (220, 311, 111) and its binding energy with Li_2S_4.

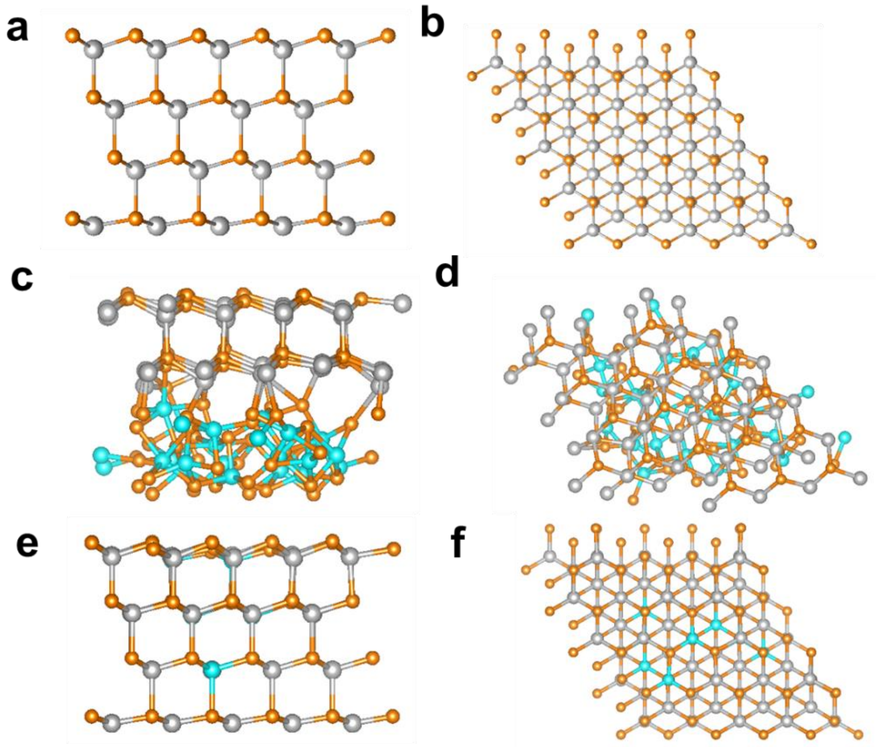

Figure S2. The structures of a,b) ZnSe, c,d)$CoSe_2$/ZnSe, and e,f) $Co_{0.125}Zn_{0.875}Se$.

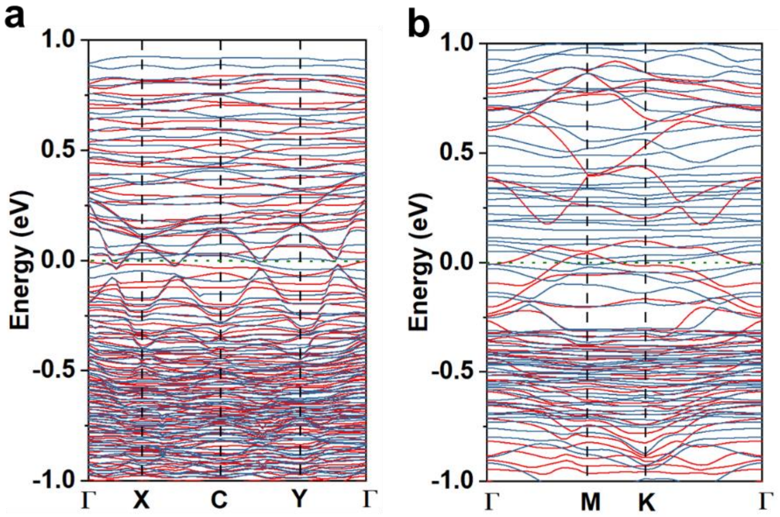

Figure S3. The band structure of a) $CoSe_2$/ZnSe, and b) $Co_{0.125}Zn_{0.875}Se$.

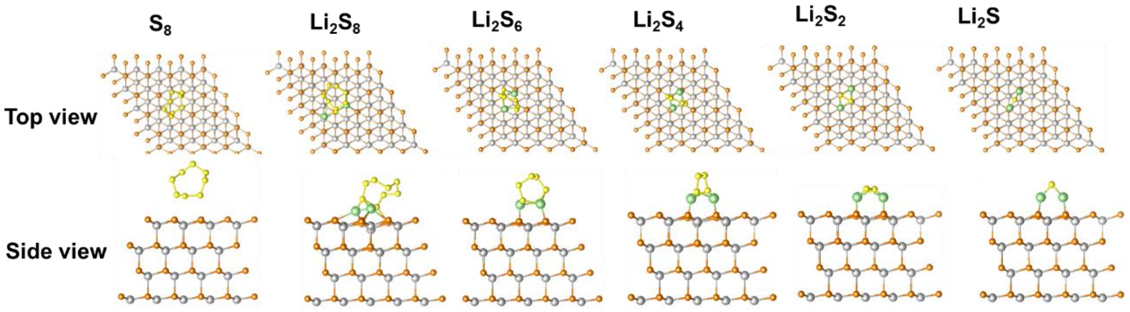

Figure S4. The optimized adsorption configurations of S_8 and various LiPSs (Li_2S_x, x=8, 6, 4, 2, 1) on ZnSe surfaces.

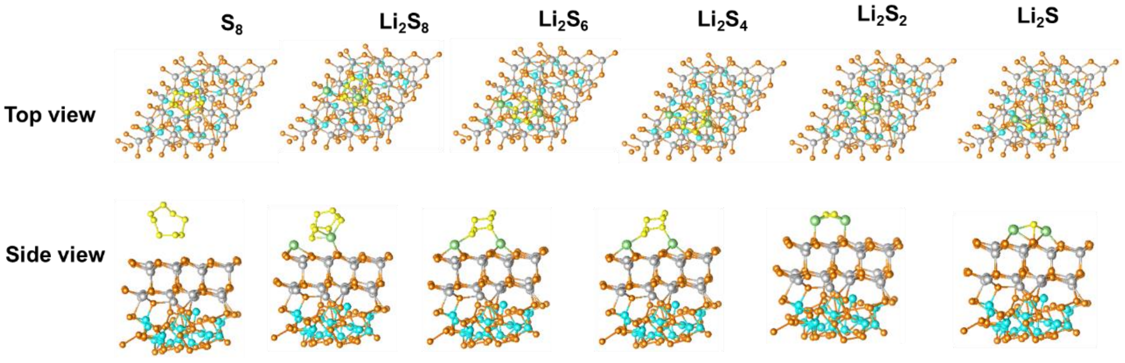

Figure S5. The optimized adsorption configurations of S_8 and various LiPSs (Li_2S_x, x=8, 6, 4, 2, 1) on $CoSe_2$/ZnSe surfaces.

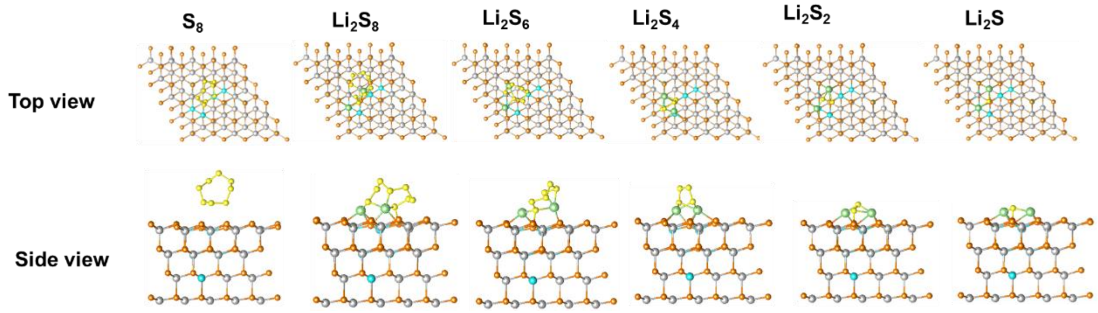

Figure S6. The optimized adsorption configurations of S_8 and various LiPSs (Li_2S_x, x=8, 6, 4, 2, 1) on $Co_{0.125}Zn_{0.875}Se$ surfaces.

$S_8^* + 2\ Li^+ + 2\ e^- = Li_2S_8^*$ (S1)

$Li_2S_8^* + 2\ Li^+ + 2\ e^- = Li_2S_6^* + Li_2S_2$ (S2)

$Li_2S_6^* + 2\ Li^+ + 2\ e^- = Li_2S_4^* + Li_2S_2$ (S3)

$Li_2S_4^* + 2\ Li^+ + 2\ e^- = Li_2S_2^* + Li_2S_2$ (S4)

$Li_2S_2^* + 3\ Li_2S_2 + 8\ Li^+ + 8\ e^- = Li_2S^* + 7\ Li_2S$ (S5)

Figure S7. Two-electrons reaction equations of Li-S batteries.

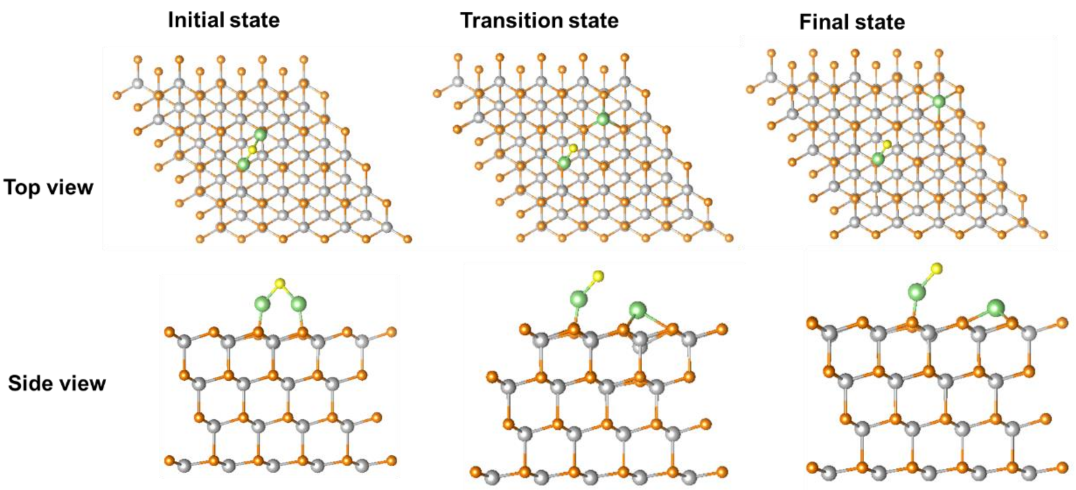

Figure S8. The decomposition of Li_2S and lithium-ion diffusion pathways on ZnSe surfaces.

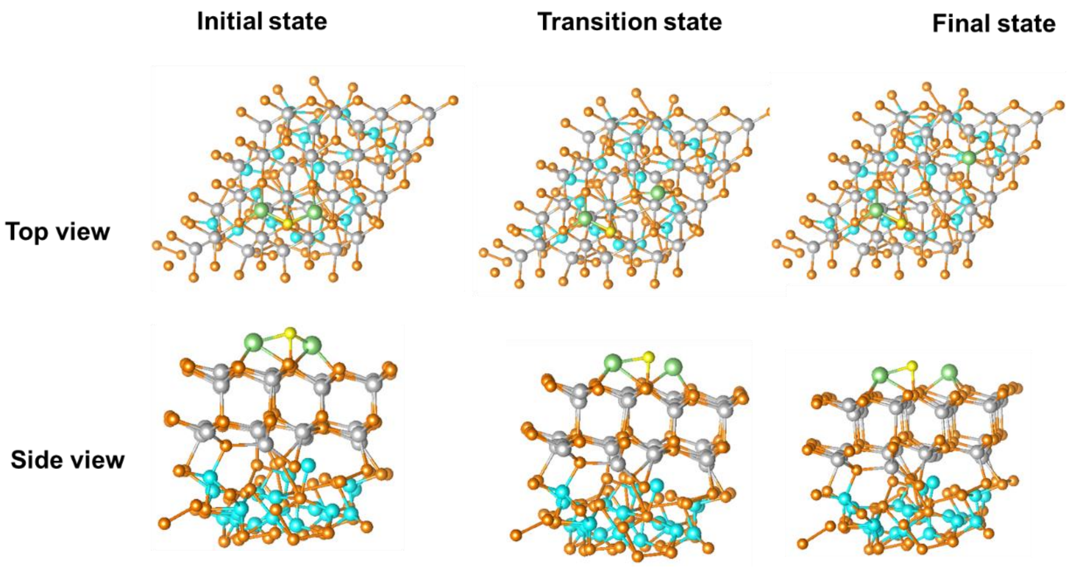

Figure S9. The decomposition of Li_2S and lithium-ion diffusion pathways on $CoSe_2$/ZnSe surfaces.

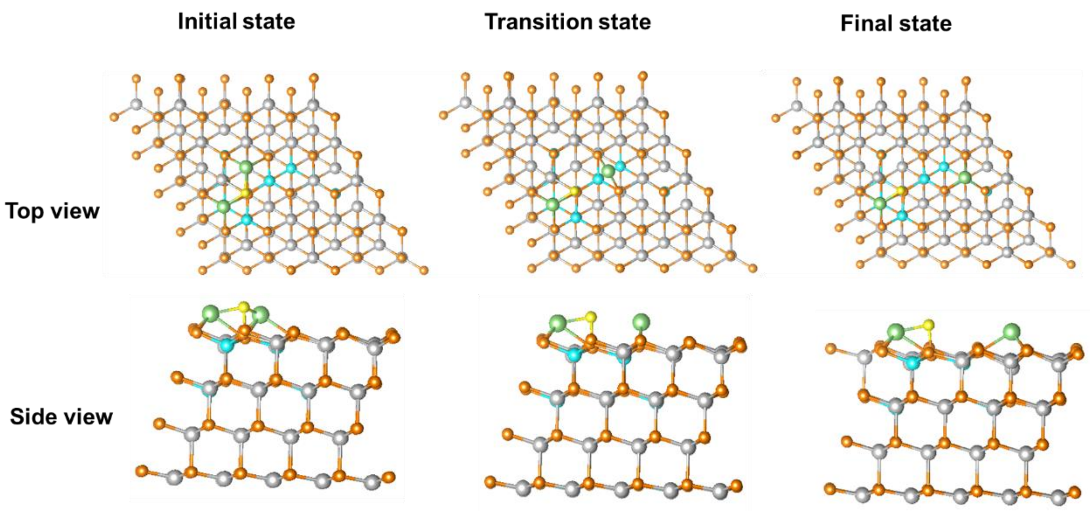

Figure S10. The decomposition of Li_2S and lithium-ion diffusion pathways on $Co_{0.125}Zn_{0.875}Se$ surfaces.

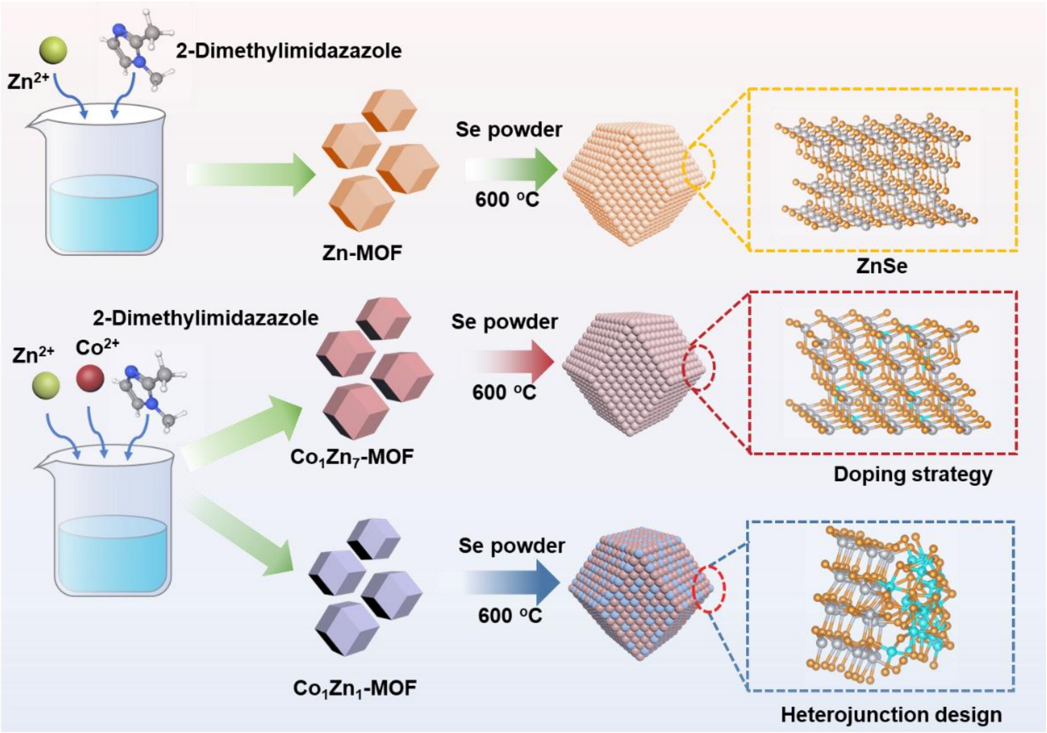

Figure S11. Schematic illustrations of the fabrication of ZnSe, $CoSe_2$/ZnSe and $Co_{0.125}Zn_{0.875}Se$.

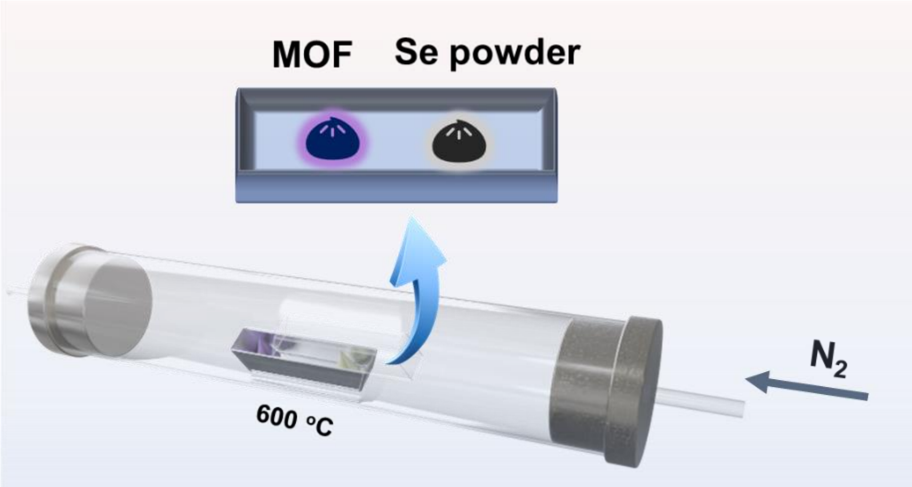

Figure S12. Schematic illustrations of the calcination process.

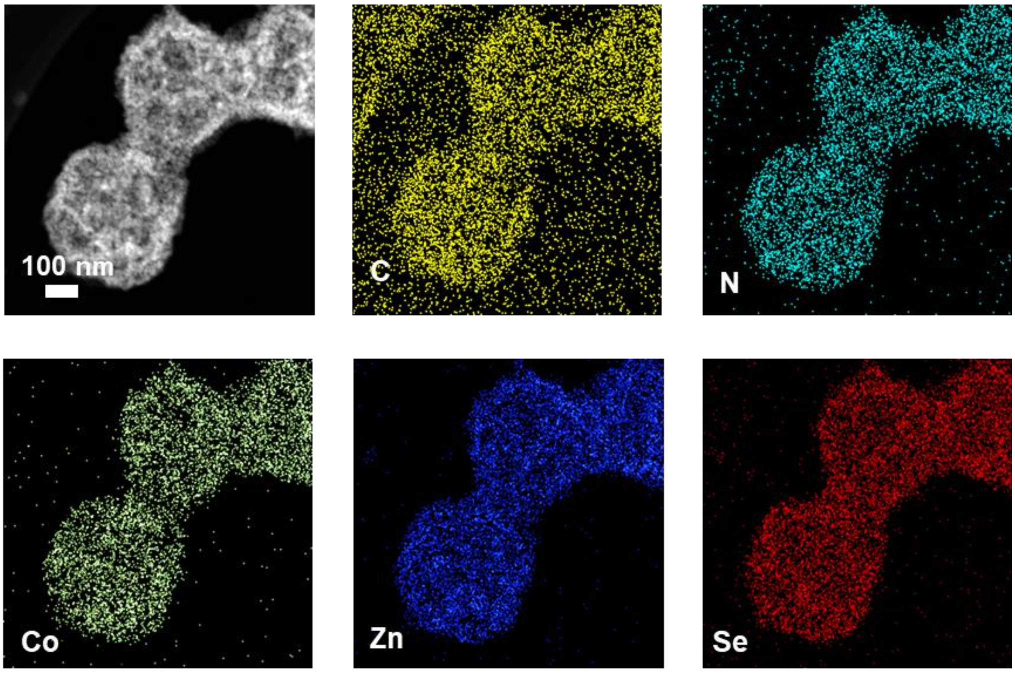

Figure S13. EDS elemental mapping of C, N, Co, Zn, and Se of $CoSe_2$/ZnSe.

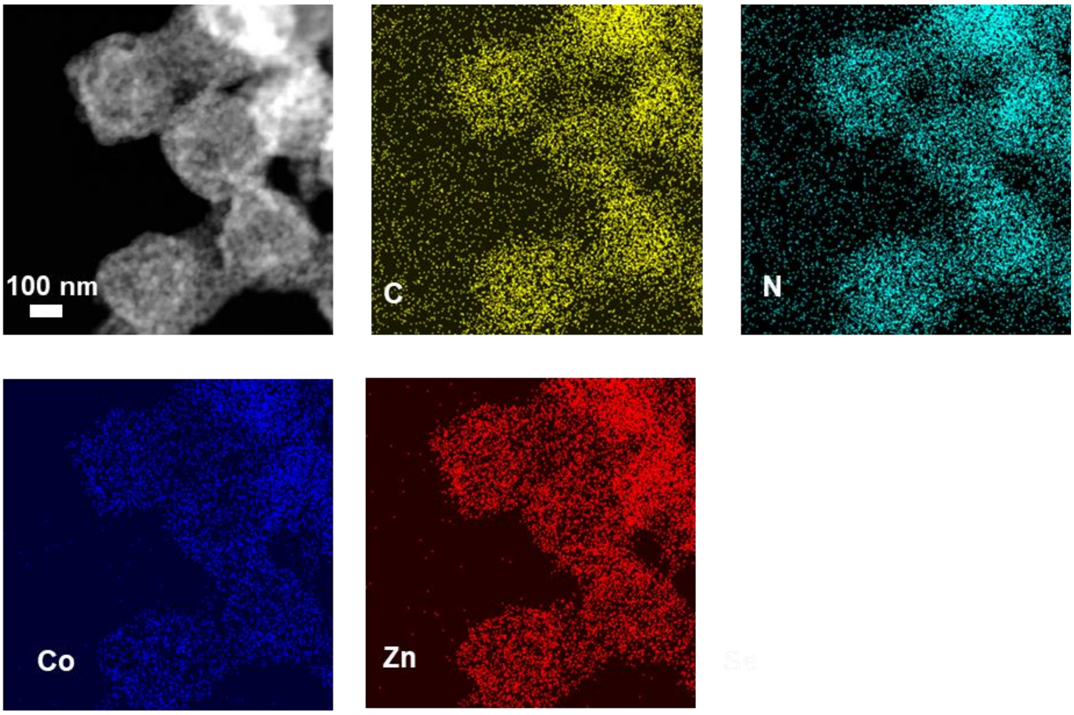

Figure S14. EDS elemental mapping of C, N, Zn, and Se of ZnSe.

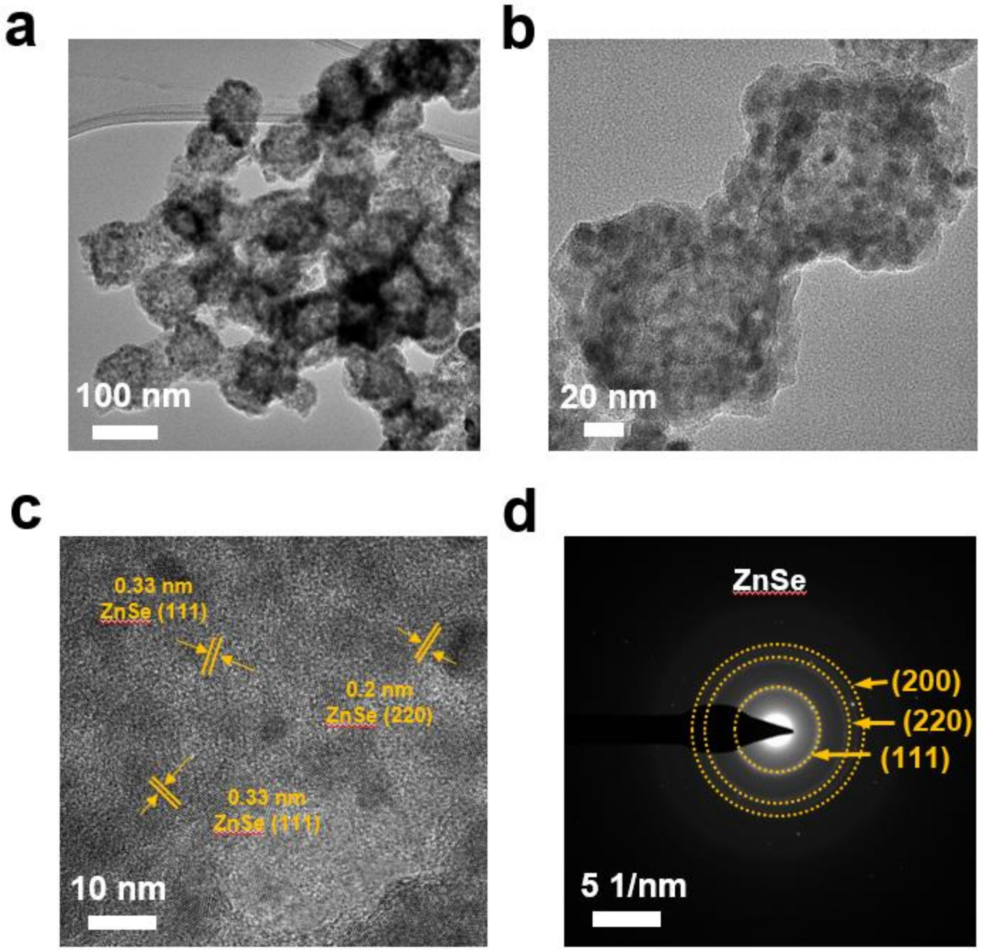

Figure S15. a,b) TEM images, c) high-resolution TEM image, d) The IFFT lattice images of the selected area of ZnSe.

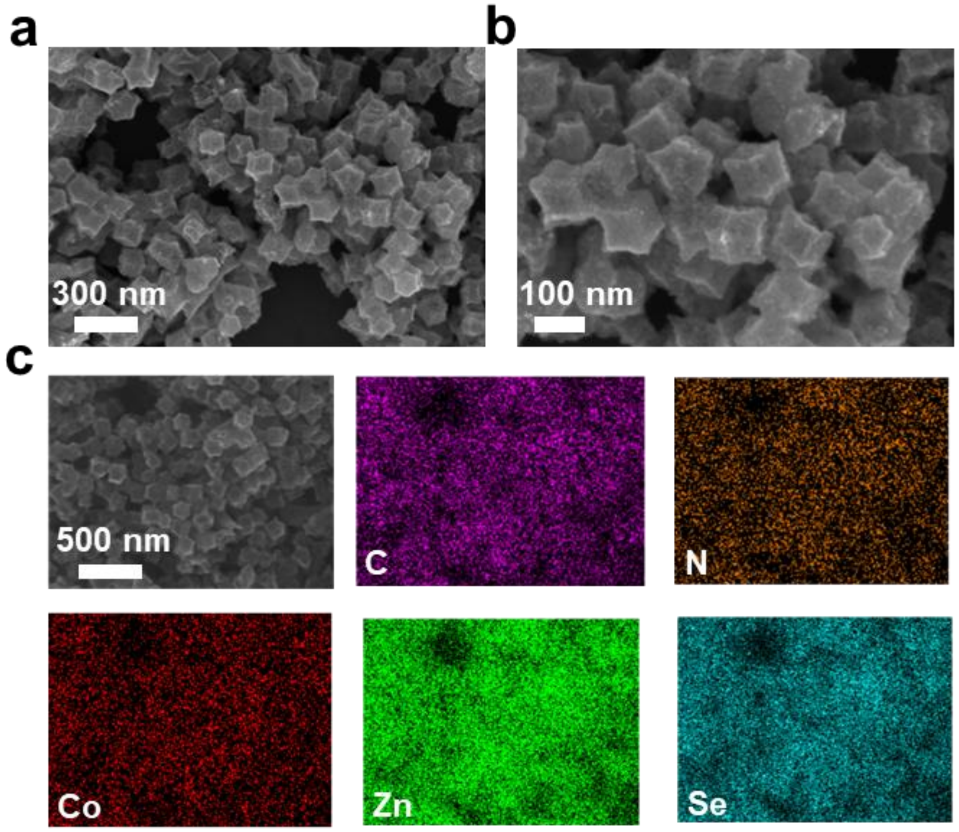

Figure S16. a, b) SEM images of $Co_{0.05}Zn_{0.95}Se_2$. c) Corresponding EDS elemental mapping of C, N, Co, Zn, and Se of the $Co_{0.05}Zn_{0.95}Se_2$.

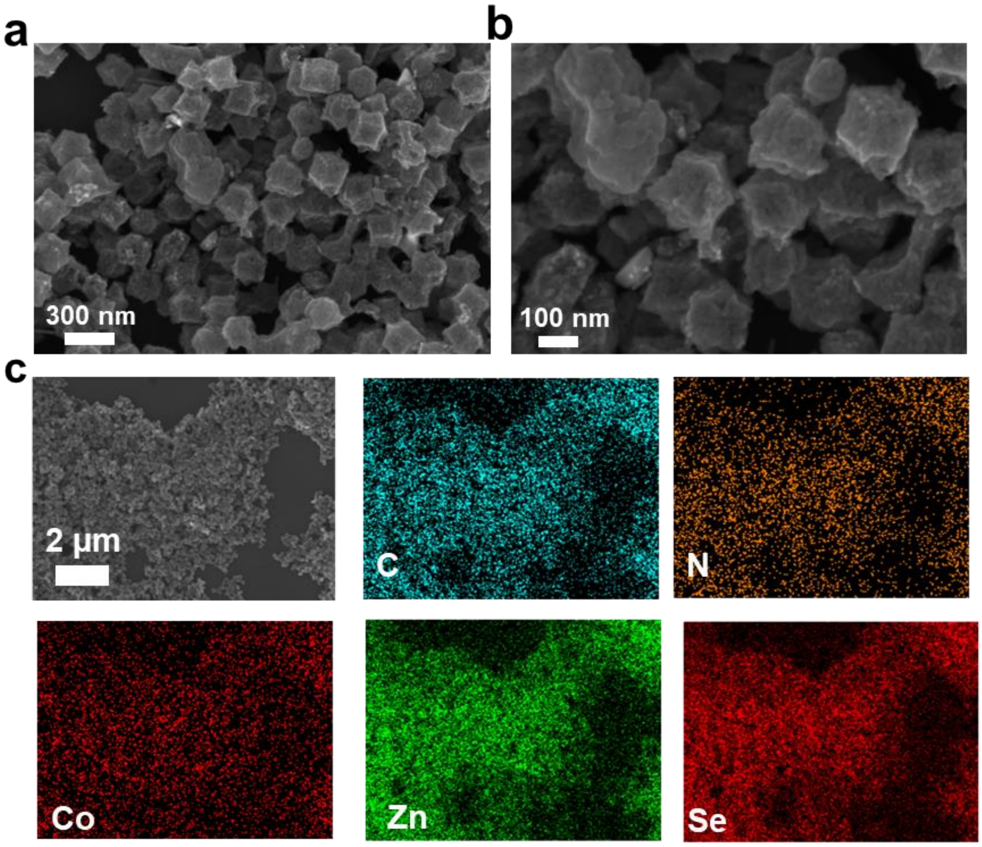

Figure S17. a, b) SEM images of $Co_{0.2}Zn_{0.8}Se_2$. c) Corresponding EDS elemental mapping of C, N, Co, Zn, and Se of the $Co_{0.2}Zn_{0.8}Se_2$.

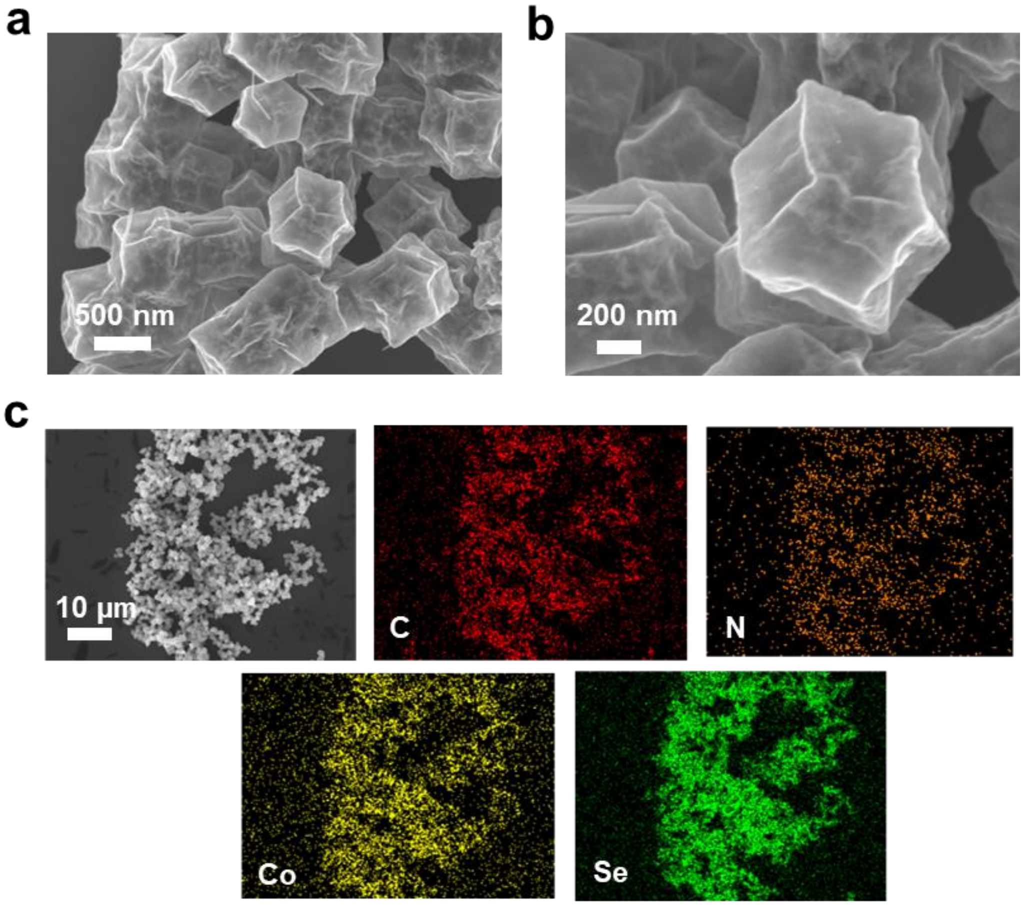

Figure S18. a-b) SEM images of $CoSe_2$. c) Corresponding EDS elemental mapping of C, N, Co, and Se of the $CoSe_2$.

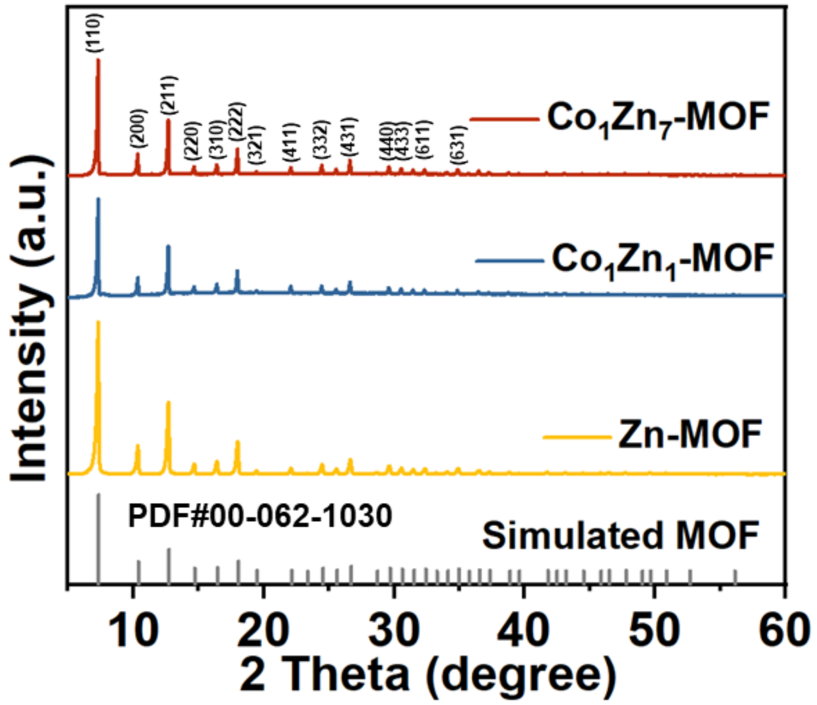

Figure S19. XRD patterns of Zn-MOFs and CoZn-MOFs.

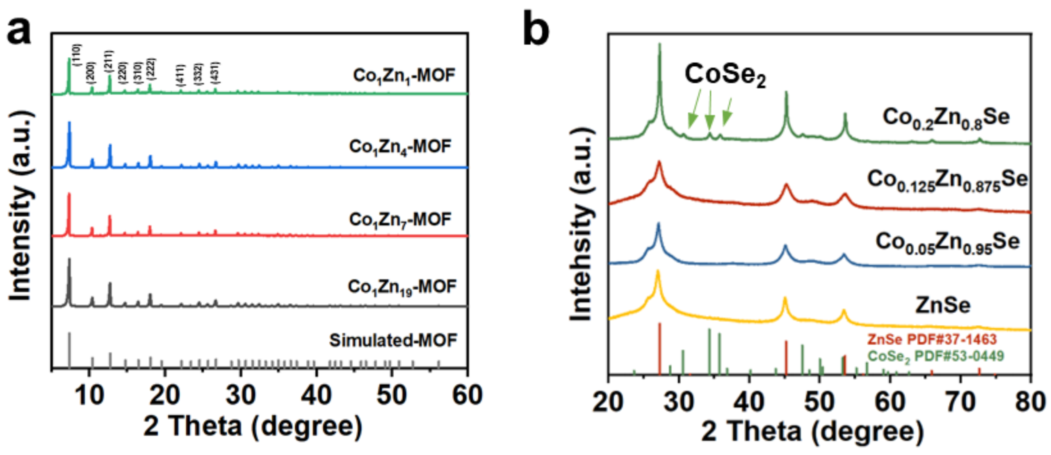

Figure S20. XRD patterns of a) Co_xZn_y-MOFs and b) $Co_xZn_{1-x}Se$ (x = Co doping concentration).

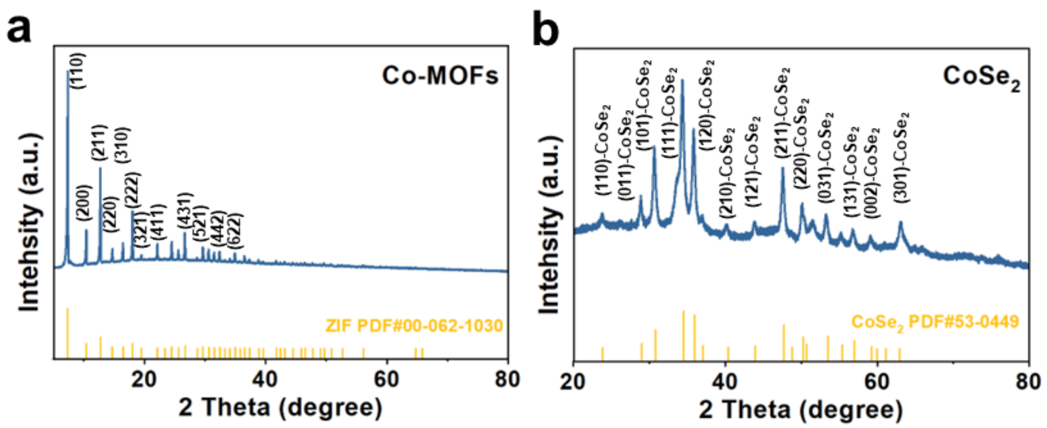

Figure S21. XRD patterns of a) Co-MOF, and b) $CoSe_2$.

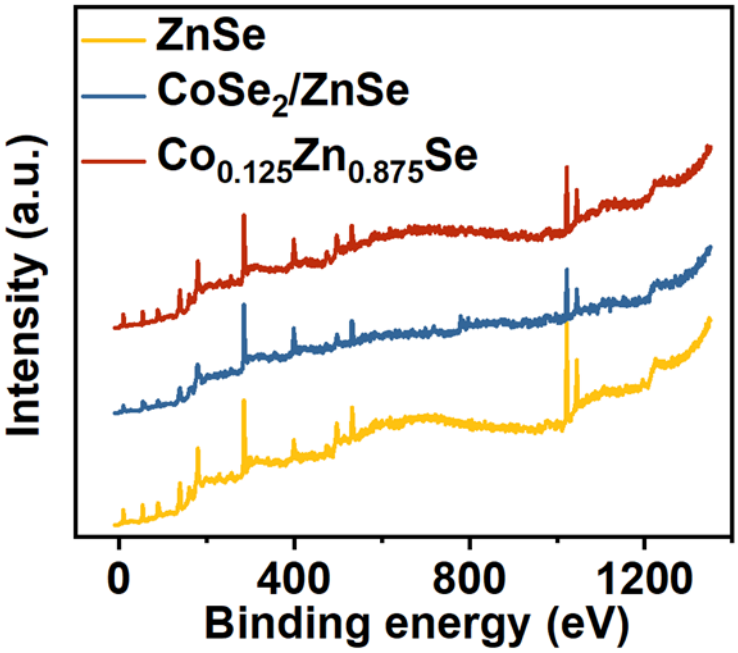

Figure S22. X-ray photoelectron spectroscopy (XPS) wide scan spectra of ZnSe, $CoSe_2$/ZnSe, and $Co_{0.125}Zn_{0.875}Se$.

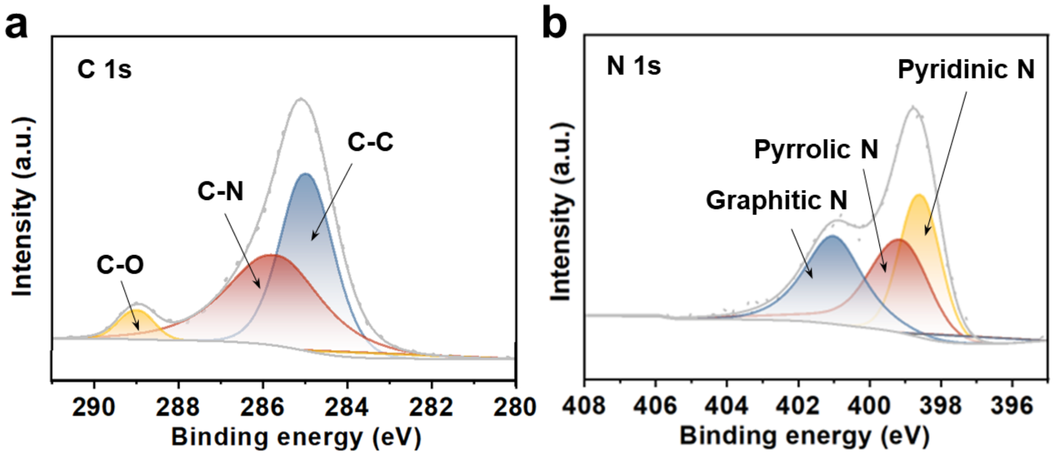

Figure S23. High-resolution X-ray photoelectron spectroscopy (XPS) spectra of a) C1s and b) N 1s of ZnSe.

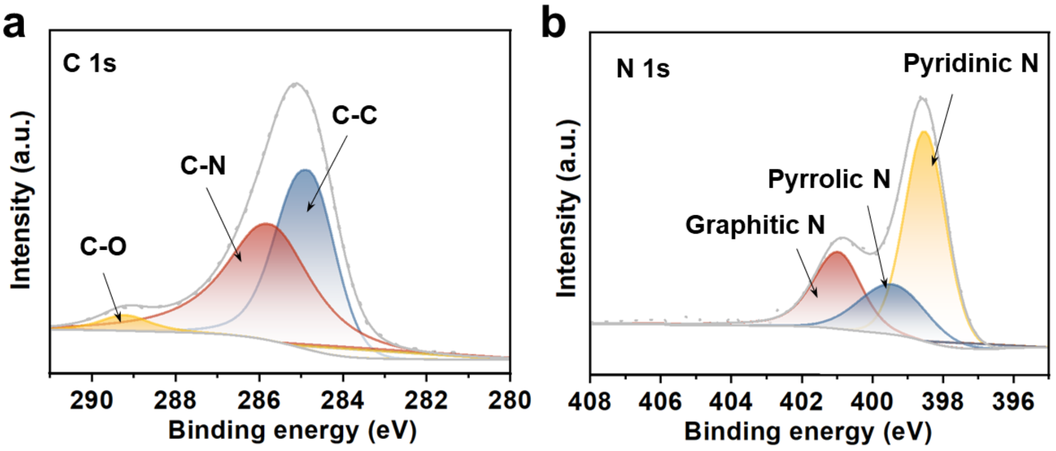

Figure S24. High-resolution XPS spectra of a) C1s and b) N 1s of $CoSe_2$/ZnSe.

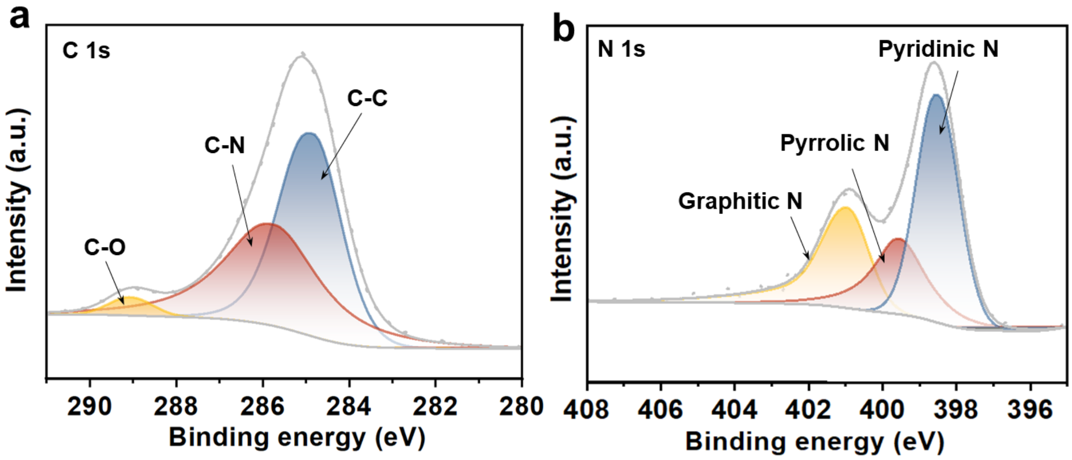

Figure S25. High-resolution XPS spectra of a) C1s and b) N 1s of $Co_{0.125}Zn_{0.875}Se$.

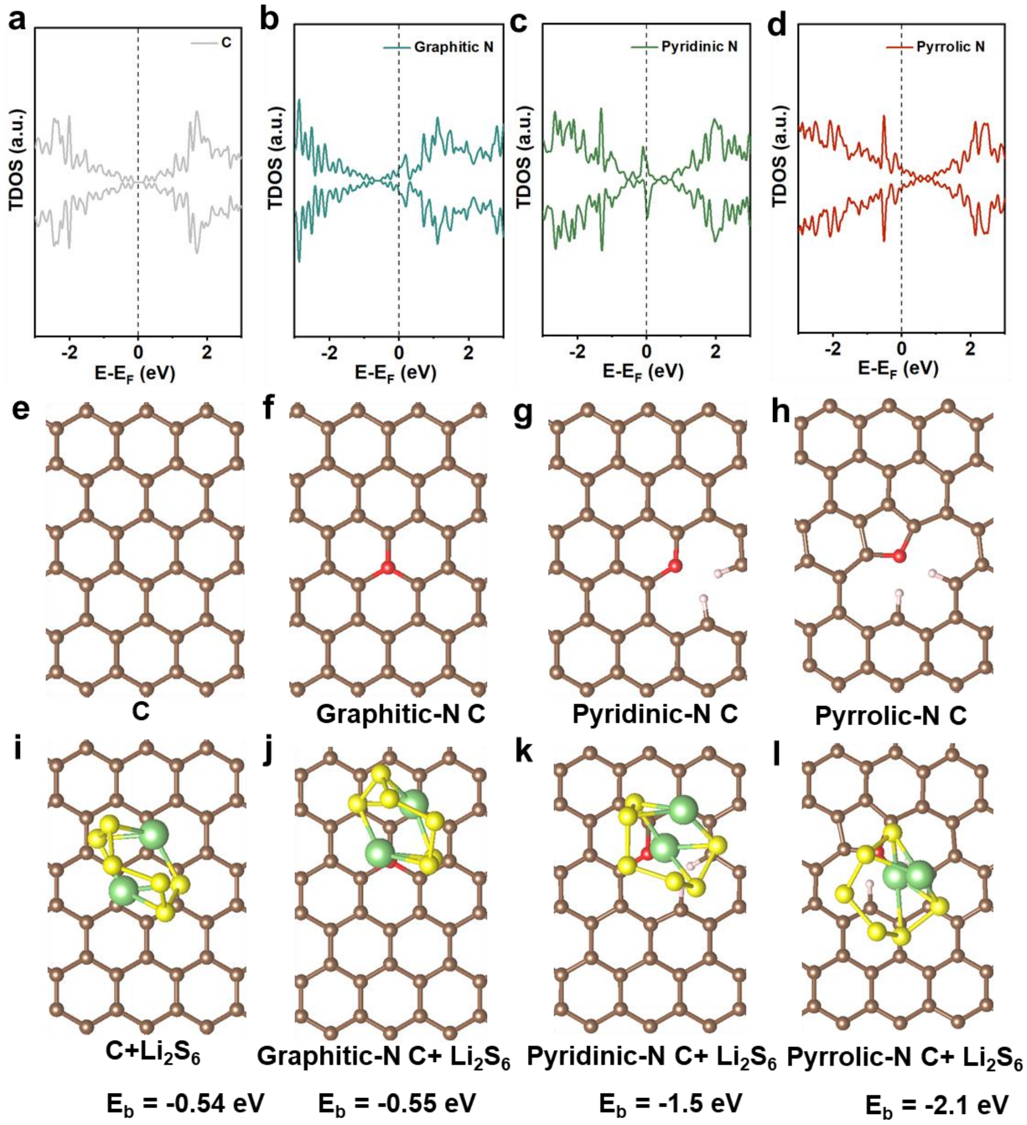

Figure S26. The density of states of a) C, b) graphitic N doping C, c) pyridinic N doping C, d) pyrrolic N doping C. The optimized structural configurations of e) C, f) graphitic N doping C, g) pyridinic N doping C, h) pyrrolic N doping C. The density states of i) C, j) graphitic N doping C, k) pyridinic N doping C, l) pyrrolic N doping C after adsorbing Li_2S_6.

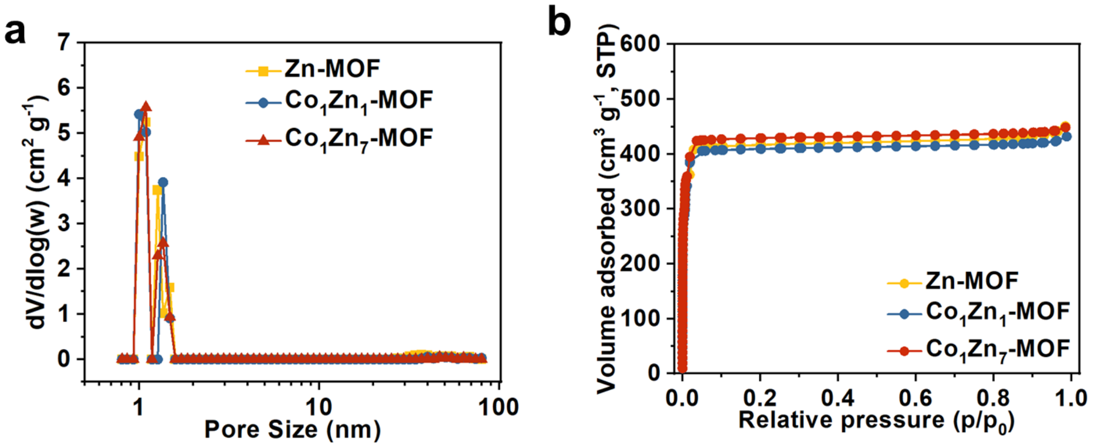

Figure S27. a) N_2 adsorption isotherms and b) pore size distribution of Zn-MOFs and CoZn-MOFs.

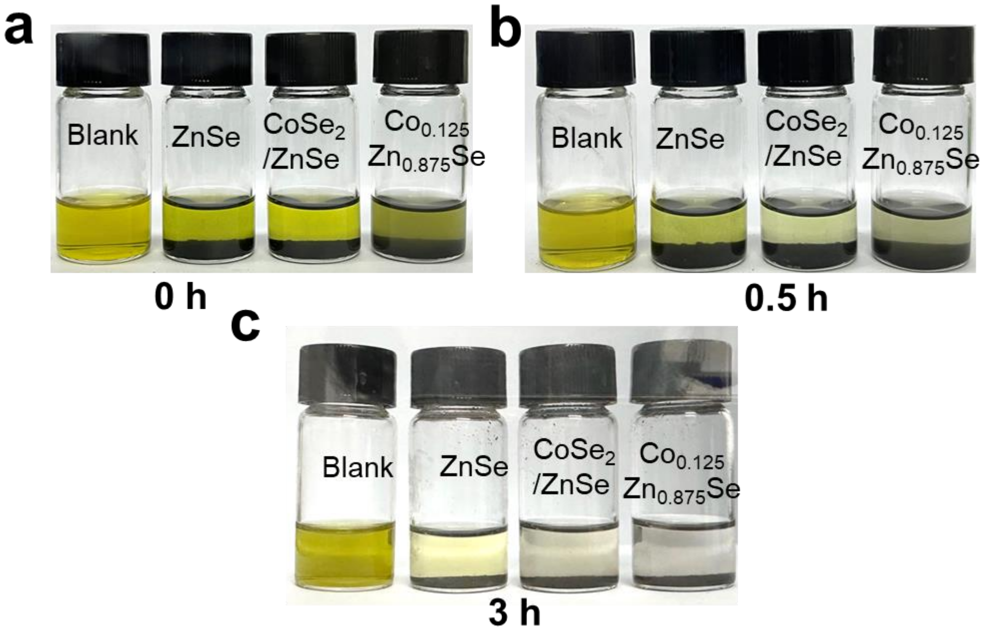

Figure S28. The Optical photographs of Li_2S_6 solutions after adsorption for blank, ZnSe, $CoSe_2$/ZnSe, and $Co_{0.125}Zn_{0.875}Se$, respectively: initial (a), after 30 min (b), and after 3 h (c).

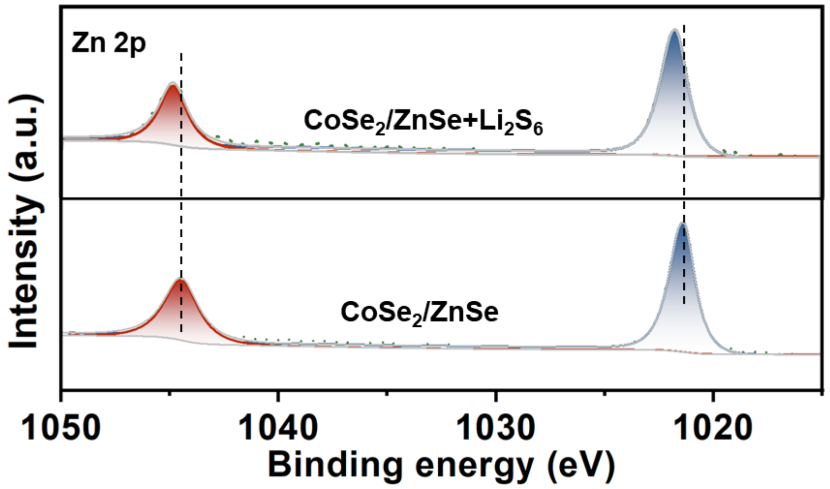

Figure S29. Zn 2p High-resolution XPS fine spectra of $CoSe_2$/ZnSe before and after Li_2S_6 adsorption.

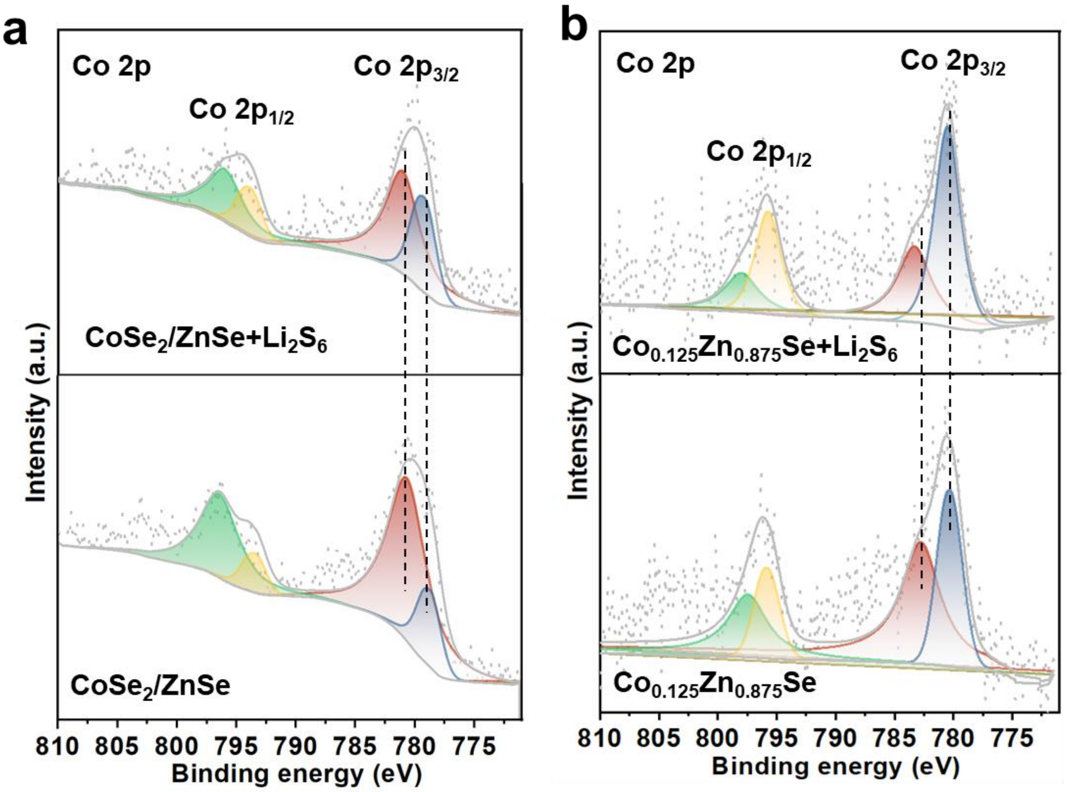

Figure S30. Co 2p High-resolution XPS fine spectra of a) $CoSe_2$/ZnSe and b) $Co_{0.125}Zn_{0.875}Se$ before and after Li_2S_6 adsorption.

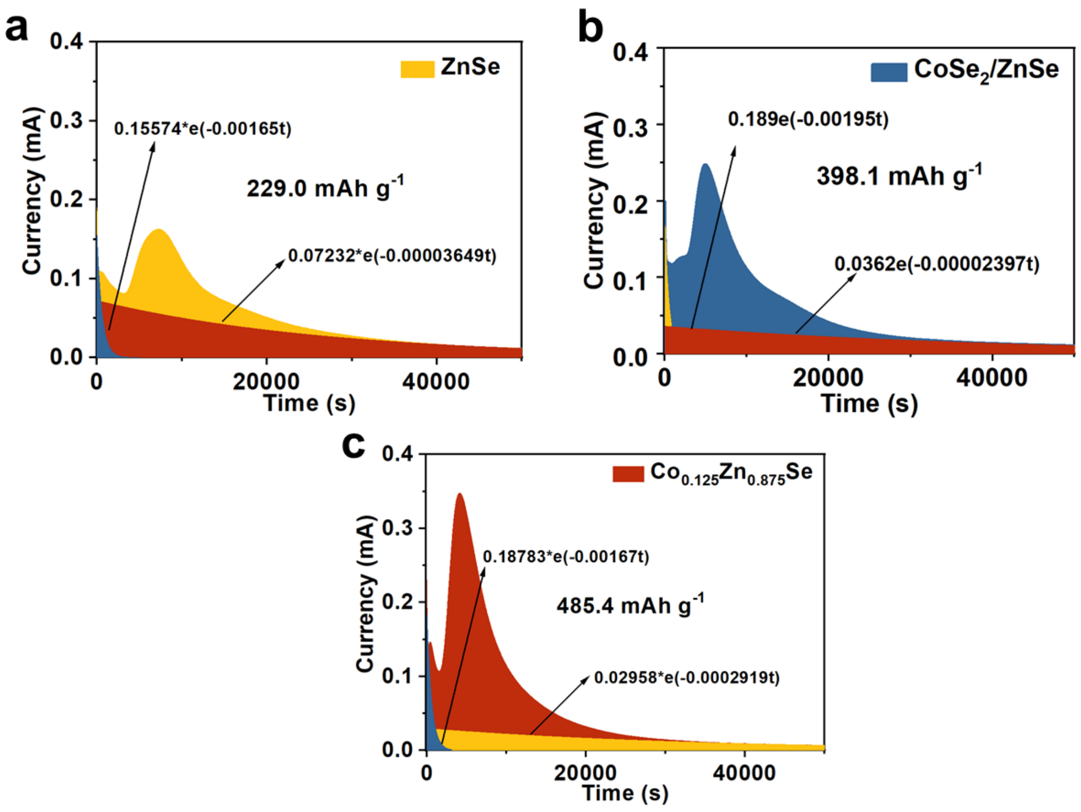

Figure S31. A current versus time analysis was conducted during potentiostatic discharge at 2.05 V across three different surfaces: a) ZnSe, b) $CoSe_2$/ZnSe, and c) $Co_{0.125}Zn_{0.875}Se$ surfaces. This experiment aimed to investigate the growth rates of Li_2S on these distinct surfaces during potentiostatic discharge at 2.05 V. Initially, the batteries underwent galvanostatic discharge to 2.06 V, following which they were held at a potentiostatic state of 2.05 V to facilitate the nucleation of Li_2S. Faraday's Law served as the basis for data fitting. The resulting current versus time curve was fitted using the integration of two exponentially decaying curves, each representing the reduction kinetics of Li_2S_6 and Li_2S_8, respectively.

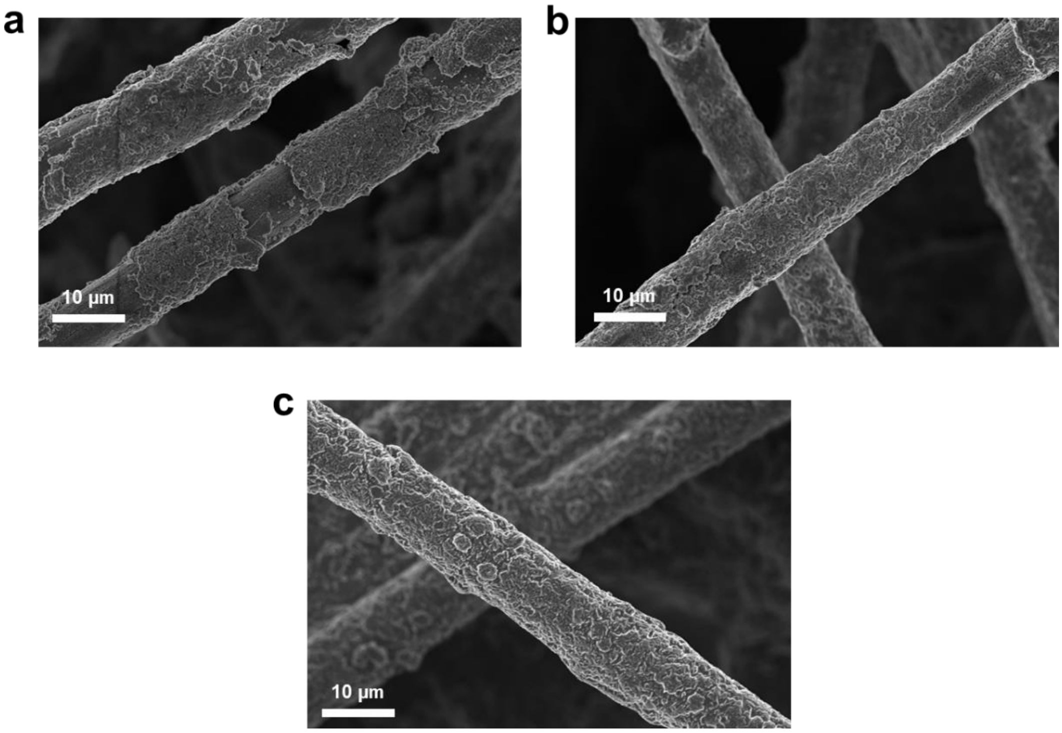

Figure S32. SEM images of SEI morphology on the surface of lithium anode in Li-S cells with different catalysts at 0.5 C after 100 cycles, a) PP separator, b) ZnSe-modified separator, c) $CoSe_2$/ZnSe-modified separator, and d) $Co_{0.125}Zn_{0.875}Se$-modified separator.

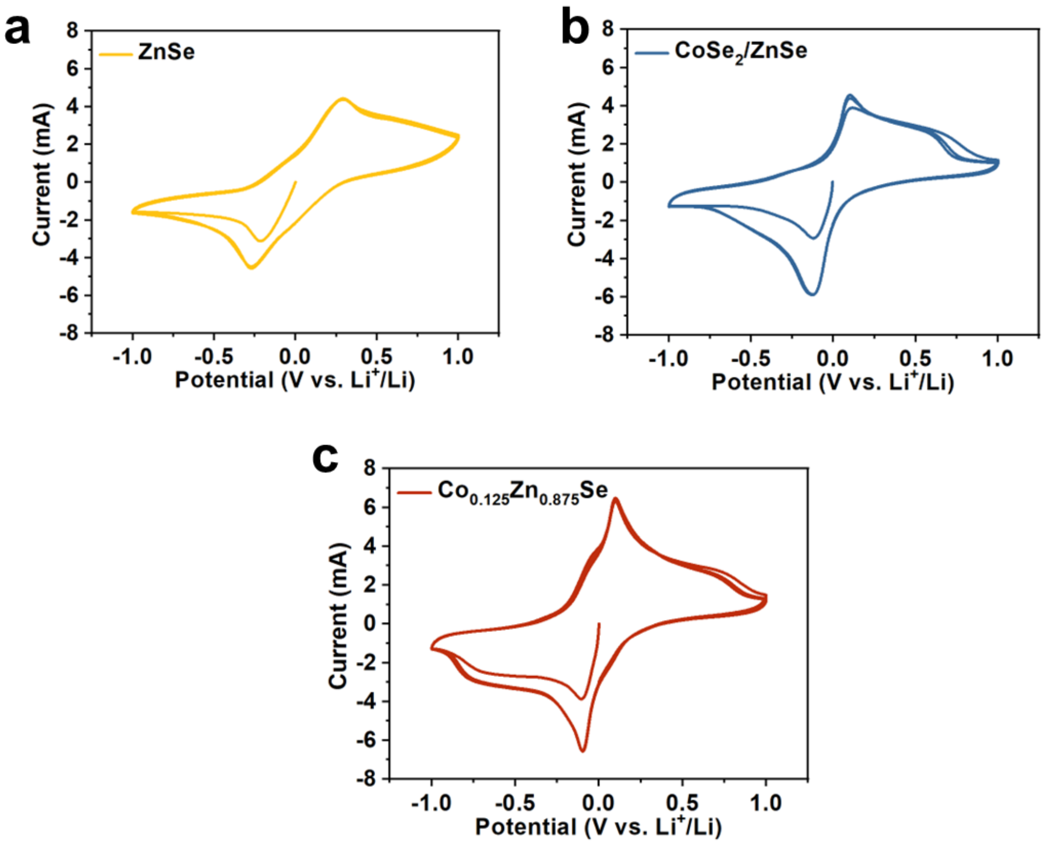

Figure S33. CV profiles of the Li_2S_6 symmetric cells assembled using a) ZnSe, b) $CoSe_2$/ZnSe, and c) $Co_{0.125}Zn_{0.875}Se$ as electrodes at a scan rate of 0.2 mV s^{-1}.

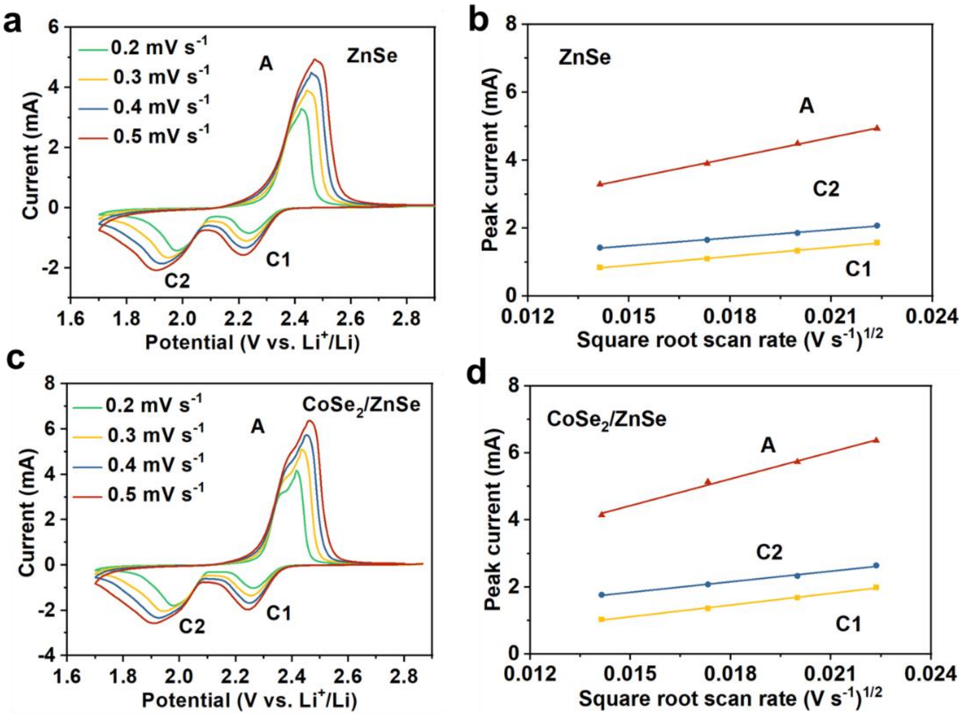

Figure S34. a) CV curves of ZnSe-based cell at different scan rates, b) The Li-ion diffusion properties of a ZnSe-based cell investigated by analyzing the CV peak currents for peaks C1, C2, and A in relation to the square root of the scan rates. c) CV curves of $CoSe_2$/ZnSe-based cell at different scan rates, d) The Li-ion diffusion properties of a $CoSe_2$/ZnSe-based cell investigated by analyzing the CV peak currents for peaks C1, C2, and A in relation to the square root of the scan rates.

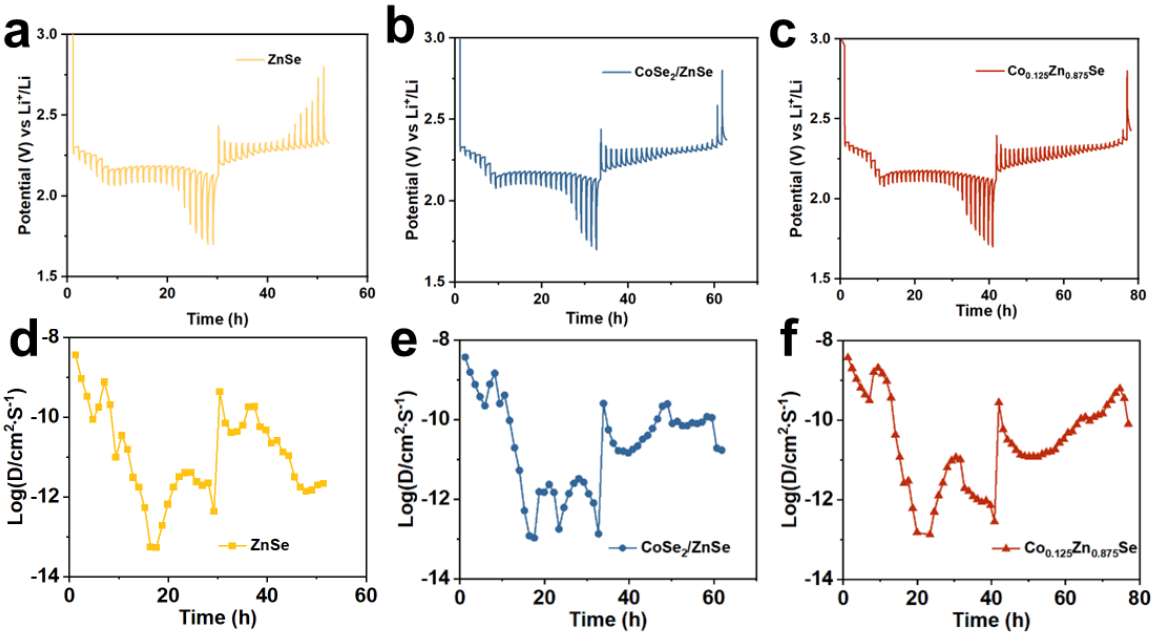

Figure S35. The GITT profiles of a) ZnSe, b) $CoSe_2$/ZnSe, and c) $Co_{0.125}Zn_{0.875}Se$. Calculated Li^+ ion diffusion coefficient during discharging and charging processes of d) ZnSe, e) $CoSe_2$/ZnSe, and f) $Co_{0.125}Zn_{0.875}Se$.

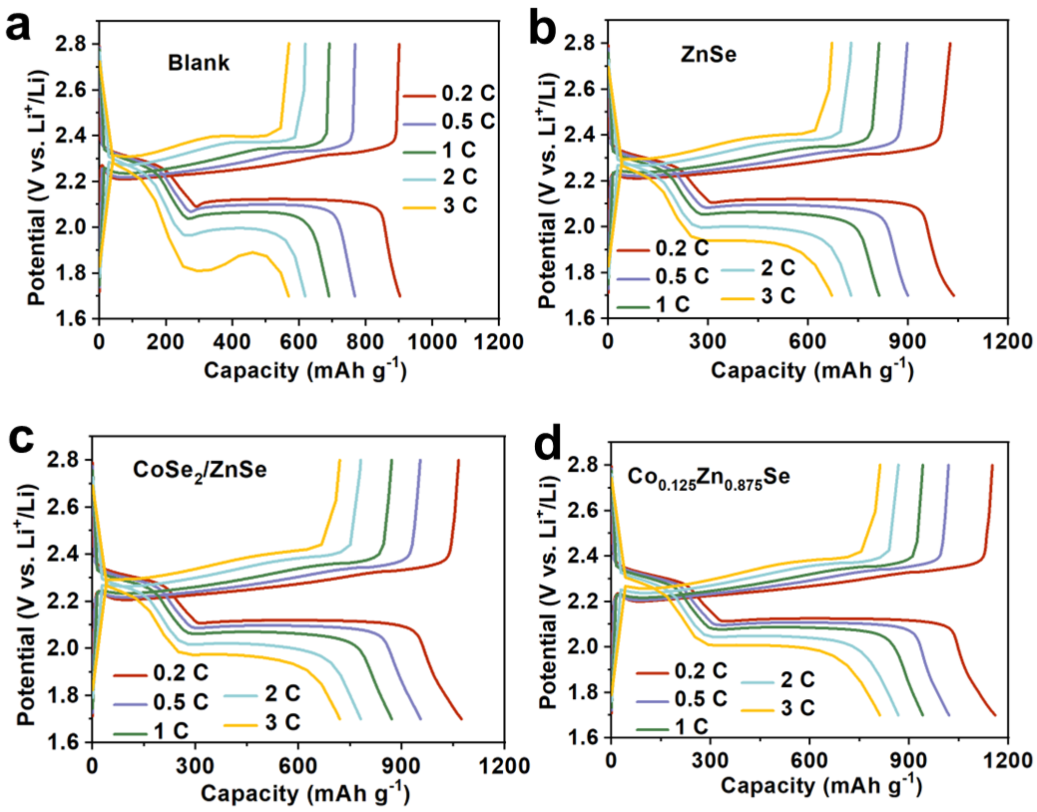

Figure S36. The galvanostatic charge–discharge curves of Li-S cells assembled using a) blank pp, b) ZnSe, c) $CoSe_2$/ZnSe, and d) $Co_{0.125}Zn_{0.875}Se$ catalysts under different rates.

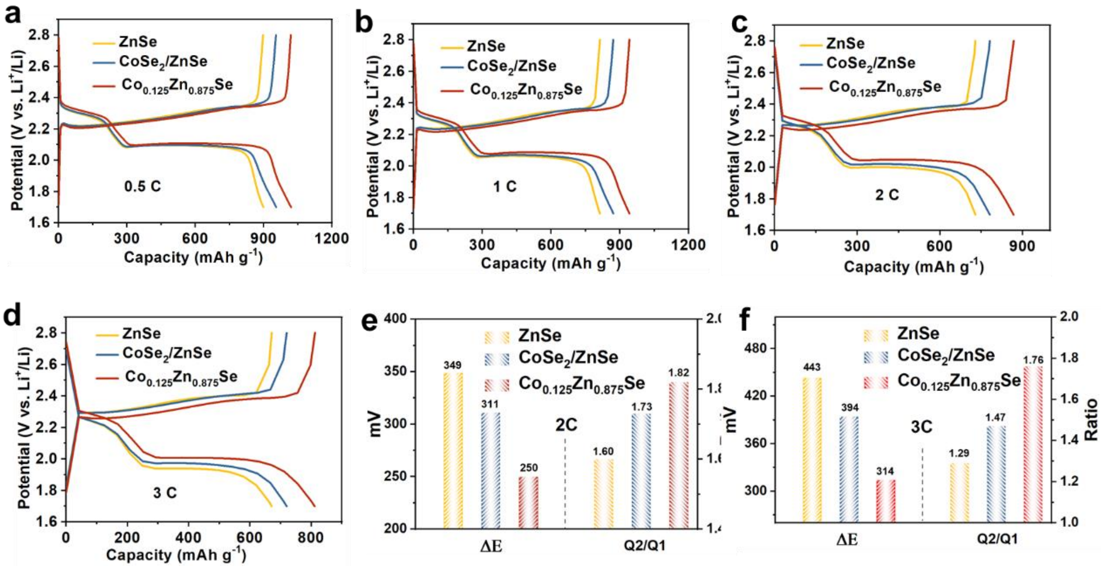

Figure S37. The galvanostatic charge–discharge curves of Li-S cells assembled using ZnSe, $CoSe_2$/ZnSe, and $Co_{0.125}Zn_{0.875}Se$ catalysts under a) 0.5 C, b) 1 C, c) 2 C, and d) 3 C. ΔE values obtained from galvanostatic charge–discharge curves of various catalysts under e) 2 C and f) 3 C.

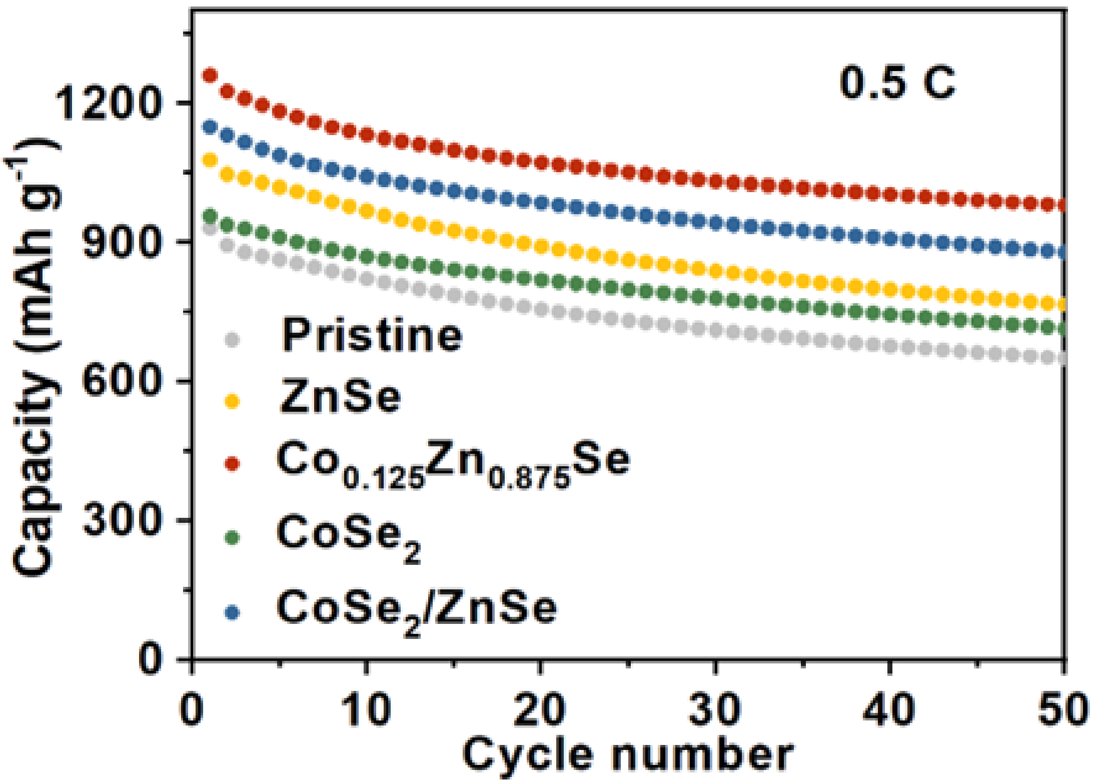

Figure S38. Cycling performance of $CoSe_2$ and various catalysts-based Li-S batteries at 0.5 C.

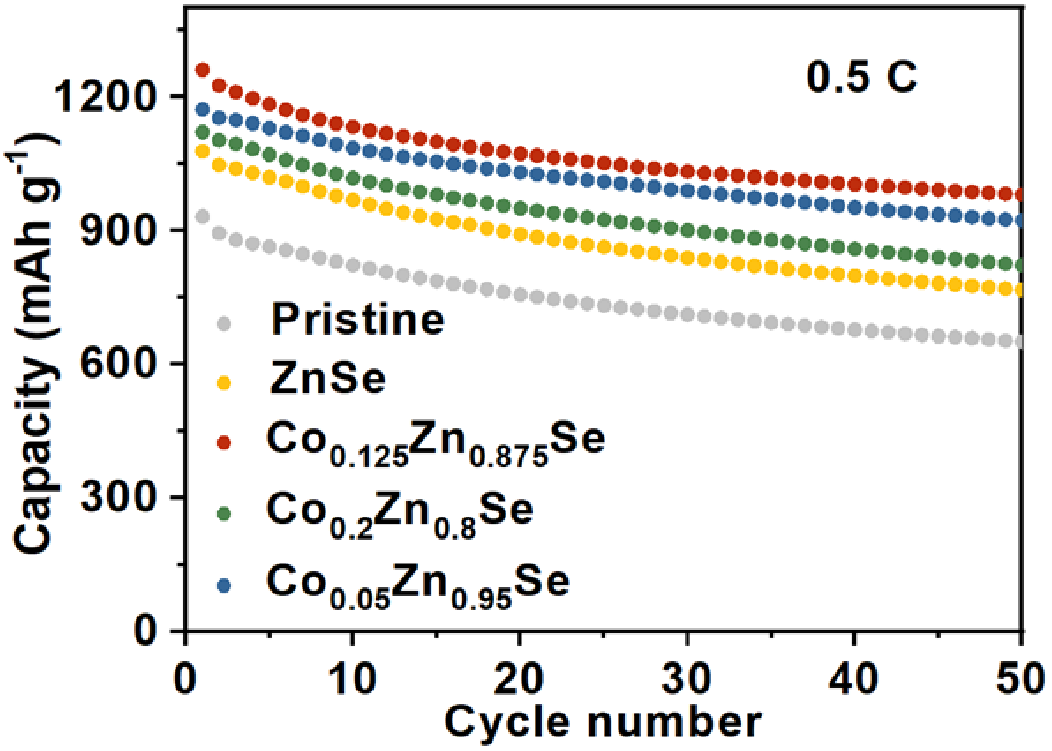

Figure S39. Cycling performance of ZnSe and $Co_xZn_{1-x}Se$ catalysts-based Li-S batteries at 0.5 C (x = Co doping concentration).

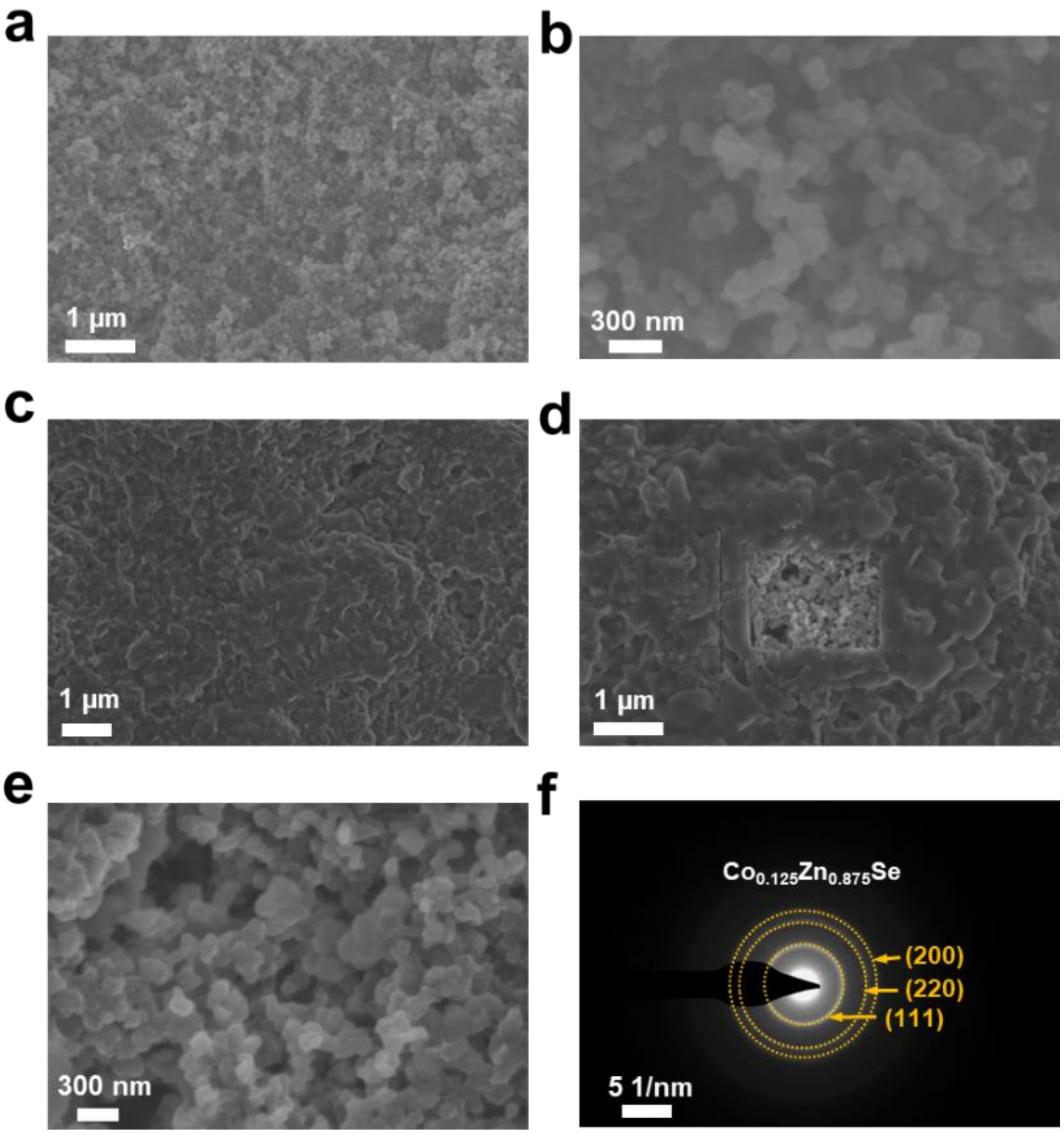

Figure S40. a, b) SEM images of the surface of $Co_{0.125}Zn_{0.875}Se_2$ coating layer before cycling. c-e) SEM images of the surface of $Co_{0.125}Zn_{0.875}Se_2$ coating layer after cycling. f) The IFFT lattice image of the $Co_{0.125}Zn_{0.875}Se_2$ after cycling.

Table S1. The adsorption energy between polysulfide and various catalysts.

	**Li_2S_8**	**Li_2S_6**	**Li_2S_4**	**Li_2S_2**	**Li_2S**
**ZnSe**	1.35	0.89	1.6	2.57	2.61
**$CoSe_2$/ZnSe**	2.44	1.37	2.05	3.05	3.96
**$Co_{0.125}Zn_{0.875}Se$**	2.99	2.01	2.18	3.37	4.39

Table S2. BET and Pore Volume of the samples.

**Sample**	**Surface area (m^2 g^{-1})**	**Pore volume (cm^3 g^{-1})**
Zn-MOF	1303	0.699
Co_1Zn_1-MOFs	1284	0.67
Co_1Zn_7-MOFs	1350	0.696
ZnSe	98	0.25
$CoSe_2$/ZnSe	69	0.23
$Co_{0.125}Zn_{0.875}Se$	127	0.34

Table S3. The diffusion coefficient of Li-ion calculated from the CV redox peaks.

	**D_{Li}^+ at peak C1 ($cm^2\ s^{-1}$)**	**D_{Li}^+ at peak C2 ($cm^2\ s^{-1}$)**	**D_{Li}^+ at peak A ($cm^2\ s^{-1}$)**
ZnSe	$1.055\ x10^{-8}$	$0.837\ x10^{-8}$	$5.527\ x10^{-8}$
$CoSe_2$-ZnSe	$1.825\ x10^{-8}$	$1.488\ x10^{-8}$	$9.303\ x10^{-8}$
$Co_{0.125}Zn_{0.875}Se$	$2.101\ x10^{-8}$	$3.168\ x10^{-8}$	$1.102\ x10^{-7}$

Table S4. Comparison of electrochemical performance of Co/$Co_{0.85}Se$@NC for Li-S batteries with present state-of-the-art catalyst materials.

Electrocatalyst	(Low rate) Cycle number	Reversible capacity (mAh g^{-1})	(High rate) Cycle number	Decay rate (%)	(High rates) Capacity (mAh g^{-1})	Highest area capacity (mAh cm^{-2})	Ref
$Co_{0.125}Zn_{0.875}Se$	(0.5C) 100	908	(2C) 1000	0.048	(3C) 828	7.6	This work
ZnCo-MOF	(0.5C) 300	688	(0.5C) 300	0.048	(2C) 552		S[9]
SeVs-$MoSe_2$	(0.5C) 200	~970	(2C) 400	0.05	(3C) 784.3	6.9	S[10]
CNT-CoP-Vp	(0.2C) 40	~1000	(2C) 300	0.083	(3C) 738	7.7	S[11]
CoFe PBA	(0.12C) 100	1143	(1.2C) 1000	0.052	(3C) 811	4	S[12]
Co@NC			(1C) 500	0.055	(3C) 741	8.17	S[13]
$W_{0.02}$-Co_3O_4			(1C) 500	0.052	(2C) 741	4.59	S[14]
$CoSe_2$/Co_3O_4@NC-CNT	(0.2C) 200	881	(2C) 500	0.045	(2C) 778	5.5	S[15]
B/2D MOF-Co	(0.5C) 200	703	(1C) 600	0.07	(2C) 590	7.8	S[16]
Co-TiN/C	(0.5C) 200	700			(2C) 752	4.7	S[17]
CC@CoSe@HPP	(0.2C) 100	~880	(2C) 1000	0.04	(3C) 754	8.1	S[18]
Co@N-HCMs	(0.2C) 100	~1000	(1C) 500	0.08	(2C) 759	7.3	S[19]
Ni/SiO_2	(0.2C) 100	922	(2C) 300	0.085	(2C) 782	2.7	S[20]
Co_3O_4-NP/N-rGO	(0.2C) 100	914	(1C) 500	0.058	(3C) 569	~4	S[21]
$Co_{0.9}Zn_{0.1}Te_2$@NC	(0.2C) 300	752	(2C) 1000	0.05	(1C) 1030	8.8	S[22]

References

[1] Z. Q. Ye, Y. Jiang, T. Y. Yang, L. Li, F. Wu, R. J. Chen, *Adv. Sci.* **2022**, *9*, 2103456.

[2] G. Kresse, J. Furthmuller, *Computational Materials Science* **1996**, *6*, 15-50.

[3] J. P. Perdew, K. Burke, M. Ernzerhof, *Phys. Rev. Lett.* **1996**, *77*, 3865-3868.

[4] S. Grimme, S. Ehrlich, L. Goerigk, *J. Comput. Chem.* **2011**, *32*, 084204.

[5] T. A. A. Batchelor, J. K. Pedersen, S. H. Winther, I. E. Castelli, K. W. Jacobsen, J. Rossmeisl, *Joule* **2019**, *3*, 834-845.

[6] W. Tang, E. Sanville, G. Henkelman, *Journal of Physics-Condensed Matter* **2009**, *21*, 084204.

[7] G. Henkelman, B. P. Uberuaga, H. Jonsson, *J. Chem. Phys.* **2000**, *113*, 9901-9904.

[8] K. Momma, F. Izumi, *J. Appl. Crystallogr.* **2011**, *44*, 1272-1276.

[9] Z. Zhu, Y. X. Zeng, Z. H. Pei, D. Y. Luan, X. Wang, X. W. Lou, *Angew. Chem. Int. Ed.* **2023**, 202305828.

[10] M. L. Wang, Z. T. Sun, H. N. Ci, Z. X. Shi, L. Shen, C. H. Wei, Y. F. Ding, X. Z. Yang, J. Y. Sun, *Angew. Chem. Int. Ed.* **2021**, *60*, 2109291.

[11] R. Sun, Y. Bai, Z. Bai, L. Peng, M. Luo, M. X. Qu, Y. C. Gao, Z. H. Wang, W. Sun, K. N. Sun, *Adv. Energy Mater.* **2022**, *12*, 2102739.

[12] Y. Chen, Y. H. Kang, H. Y. Yang, H. M. Hua, J. X. Qin, P. Liu, Y. Y. Zhang, Y. J. Zhang, J. B. Zhao, *Energy Storage Mater.* **2023**, *54*, 10047.

[13] Y. J. Li, W. Y. Wang, B. Zhang, L. Fu, M. T. Wan, G. C. Li, Z. Cai, S. B. Tu, X. R. Duan, Z. W. Seh, J. J. Jiang, Y. M. Sun, *Nano Lett.* **2021**, *21*, 1c02161.

[14] S. N. Wang, R. M. Hu, D. Yuan, L. Zhang, C. Wu, T. Y. Ma, W. Yan, R. Wang, L. Liu, X. C. Jiang, H. K. Liu, S. X. Dou, Y. H. Dou, J. T. Xu, *Carbon Energy* **2023**, *2*, 329.

[15] R. R. Chu, T. T. Nguyen, Y. Q. Bai, N. H. Kim, J. H. Lee, *Adv. Energy Mater.* **2022**, *12*, 2102805.

[16] Y. J. Li, S. Y. Lin, D. D. Wang, T. T. Gao, J. W. Song, P. Zhou, Z. K. Xu, Z. H. Yang, N. Xiao, S. J. Guo, *Adv. Mater.* **2020**, *32*, 1906722.

[17] Q. B. Liu, Y. J. Wu, D. Li, Y. Q. Peng, X. Y. Liu, B. Q. Li, J. Q. Huang, H. J. Peng, *Adv. Mater.* **2023**, *35*, 2209233.

[18] Z. Q. Ye, Y. Jiang, L. Li, F. Wu, R. J. Chen, *Adv. Mater.* **2020**, *32*, 2002168.

[19] L. Su, J. Q. Zhang, Y. Chen, W. Yang, J. Wang, Z. P. Ma, G. J. Shao, G. X. Wang, *Nano Energy* **2021**, *85*, 105981.

[20] C. Chen, Q. B. Jiang, H. F. Xu, Y. P. Zhang, B. K. Zhang, Z. Y. Zhang, Z. Lin, S. Q. Zhang, *Nano Energy* **2020**, *76*, 105033.

[21] R. J. Xiao, D. Luo, J. Y. Wang, H. Lu, H. Ma, E. M. Akinoglu, M. L. Jin, X. Wang, Y. G. Zhang, Z. W. Chen, *Adv. Sci.* **2022**, *9*, 2202352.

[22] B. Wang, L. Wang, D. Ding, Y. J. Zhai, F. B. Wang, Z. X. Jing, X. F. Yang, Y. Y. Kong, Y. T. Qian, L. Q. Xu, *Adv. Mater.* **2022**, *34*, 2204403.
